# Propionate-Producing Consortium Restores Antibiotic-Induced Dysbiosis in a Dynamic *in vitro* Model of the Human Intestinal Microbial Ecosystem

**DOI:** 10.3389/fmicb.2019.01206

**Published:** 2019-05-31

**Authors:** Racha El Hage, Emma Hernandez-Sanabria, Marta Calatayud Arroyo, Ruben Props, Tom Van de Wiele

**Affiliations:** Center for Microbial Ecology and Technology, Ghent University, Ghent, Belgium

**Keywords:** propionate, dysbioses, consortium, human intestinal, microbiome, metabolic disease

## Abstract

Metabolic syndrome is a growing public health concern. Efforts at searching for links with the gut microbiome have revealed that propionate is a major fermentation product in the gut with several health benefits toward energy homeostasis. For instance, propionate stimulates satiety-inducing hormones, leading to lower energy intake and reducing weight gain and associated risk factors. In (disease) scenarios where microbial dysbiosis is apparent, gut microbial production of propionate may be decreased. Here, we investigated the effect of a propionogenic bacterial consortium composed of *Lactobacillus* *plantarum*, *Bacteroides* *thetaiotaomicron*, *Ruminococcus* *obeum*, *Coprococcus* *catus*, *Bacteroides* *vulgatus*, *Akkermansia* *muciniphila*, and *Veillonella* *parvula* for its potential to restore *in vitro* propionate concentrations upon antibiotic-induced microbial dysbiosis. Using the mucosal simulator of the human intestinal microbial ecosystem (M-SHIME), we challenged the simulated colon microbiome with clindamycin. Addition of the propionogenic consortium resulted in successful colonization and subsequent restoration of propionate levels, while a positive effect on the mitochondrial membrane potential (ΔΨ_m_) was observed in comparison with the controls. Our results support the development and application of next generation probiotics, which are composed of multiple bacterial strains with diverse functionality and phylogenetic background.

## Introduction

The human gut plays a major role in nutrition, metabolism, pathogen resistance, and regulation of immune response (Dethlefsen et al., 2008; Turroni et al., 2012). Microbial fermentation processes in the gut leading to production of short chain fatty acids (SCFA), are the result of metabolic interactions between different gut species (Dethlefsen et al., 2008). The main SCFA (acetate, propionate, and butyrate) (McOrist et al., 2008) perform important physiological functions (Hosseini et al., 2011), occur in molar ratio of 3:1:1 in the colon (Hosseini et al., 2011) and are used by the microbiota for growth and maintenance of cellular functions in the host (Fernandes et al., 2014). Acetate is absorbed and transported to the liver for cholesterol and fatty acid synthesis in the host, playing major role in enhancing ileal motility (Hosseini et al., 2011), while butyrate is the key energy source for colonocytes. Butyrate prevents proliferation of cancerous cells and stimulates differentiation of colon epithelial cells (Hosseini et al., 2011). The health effects of propionate go beyond the gut epithelium, as it lowers serum cholesterol levels, lipogenesis, and carcinogenesis risk (Fernandes et al., 2014). Propionate may also decrease obesity by promoting the secretion of PYY and GLP-1 hormones from human colonic cells (Tolhurst et al., 2011; Chambers et al., 2014; Psichas et al., 2014; Morrison and Preston, 2016), inducing satiety and subsequently reducing energy intake and promoting weight loss (Arora et al., 2011). Propionate is a particularly interesting metabolite in the context of the etiology and progress of metabolic syndrome, which is becoming a public health issue. Metabolic syndrome is defined as a cluster of different biological factors characterized by obesity, dyslipidemia, and type 2 diabetes (Halcox and Quyyumi, 2005; Alberti et al., 2009; Moore et al., 2017; Nolan et al., 2017). This syndrome is linked to different comorbidities like cardiovascular disease, non-alcoholic fatty liver, arthritis, chronic kidney disease, and several types of cancers (Halcox and Quyyumi, 2005; Dugas et al., 2016; Moore et al., 2017). Recent studies have reported an association between gut microbiota and metabolic syndrome, as the gut composition differs between healthy and diseased individuals. The gut microbiota is responsible for producing different regulatory peptide hormones (Silva and Bloom, 2012), depending on the nutrient supply provided, as their interaction with receptors at different points in the gut-brain axis leads to satiety (Silva and Bloom, 2012). Propionate has been reported to have the highest affinity for the free fatty acid receptor 2 (FFAR 2), involved in the regulation of metabolic homeostasis. In fact, long-term propionate delivery in the gut stimulates anorexigenic gut hormones, reducing intra-abdominal fat accretion, intrahepatocellular lipid content, and hepatic cholesterol synthesis in humans (Arora et al., 2011; Chambers et al., 2014). In addition, propionate is involved in activation of intestinal gluconeogenesis (IGN), thus regulating food intake and enhancing insulin sensitivity (Li et al., 2017).

As propionate production is associated with gut microbiome composition and functionality, different modulators such as antibiotics, prebiotics, and probiotics (El Hage et al., 2017) can impact this metabolite. Antibiotics may foster pathogenic opportunistic bacteria (Francino, 2016), influencing human health (Jin et al., 2016). Long-term antibiotic use can lead to increased body mass index and weight gain in both humans and farm animals (Francino, 2016), alter transcription of genes involved in liver lipid metabolism (Jin et al., 2016), increase insulin resistance, and steatosis in patients with fatty liver (Miquilena-Colina et al., 2011). Antibiotic use has also been reported to cause bacterial translocation, which may represent an additional inflammatory stimulus potentially promoting obesity (Knoop et al., 2015). Hence, antibiotic use is considered a risk factor for metabolic disorders (Economopoulos et al., 2016; Francino, 2016). For instance, the Cl^-^ molecule present in the clindamycin disrupts the mitochondrial membrane potential (Goldhill et al., 1996); similar disruption has been reported following oxidative damage in metabolic syndrome (Nicolson, 2007). Thus, we applied clindamycin as an agent to simulate the conditions of oxidative stress occurring during metabolic syndrome.

Synthetic microbial communities have been proposed to prevent and treat disease and reverse gut dysbiosis more effectively than single strain approaches (El Hage et al., 2017). Because of its beneficial effects on the host metabolism, propionate in our gut could contribute to solve the metabolic syndrome puzzle. We therefore aimed at engineering a propionate-producing synthetic microbial community, with functional redundancy on the different metabolic pathways for propionate production (acrylate, succinate, propanediol) (Reichardt et al., 2014). We then evaluated its potential to restore gut functionality after antibiotic-associated dysbiosis, using the Simulator of the Human Intestinal Microbial Ecosystem (SHIME). Knowledge regarding successful engraftment of functional communities can be applied for developing preventative novel probiotic strategies to ameliorate microbiome imbalances associated with metabolic syndrome.

## Materials and Methods

### Selection of Strains for the Propionate-Producing Consortium (PPC)

Gut commensal strains were selected based on the reported metabolic pathways for propionate production (succinate, acrylate, and propanediol pathways) (Reichardt et al., 2014; Table 1). *Veillonella parvula, Bacteroides vulgatus*, and *Bacteroides thetaiotaomicron* were reported to take the succinate pathway in which they use succinate to produce propionate (Louis et al., 2014). *Coprococcus catus* has been reported to take the acrylate pathway for propionate production, in which lactate is consumed (Louis et al., 2014). To provide lactate for *Coprococcus catus*, we supplied the consortium with *Lactobacillus plantarum*, a lactic acid bacterium, producing lactate and acetate that can support the acrylate pathway. *Ruminococcus obeum* uses the propanediol pathway (Louis et al., 2014) fermenting fucose for propionate production. Fucose can be produced from mucin degradation, and as *Akkermansia muciniphila* is a mucin-degrader producing propionate, this bacterium was used to enrich the propanediol pathway. The single strains produced between 0.4 and 3 mM of propionate, and the final propionate for the consortium was 34.5 mM on average (Supplementary Figure 4).

**Table 1:** Bacterial strains used to prepare the propionate-producing consortium according to the different metabolic pathways for propionate production (Acrylate, Succinate, Propanediol).

Bacterial species	Source
*Bacteroides vulgates*	LMG17767
*Bacteroides thetaiotaomicron*	DSM 2079
*Coprococcus catus*	ATCC 27761
*Veillonella parvula*	DSM 2007
*Ruminococcus obeum* (*Blautia*)	DSM 25238
*Akkermansia muciniphila*	DSM 22959
*Lactobacillus plantarum*	LMG 9211


*For Acrylate pathway, Lactobacillus plantarum and Coprococcus catus were selected; for succinate pathway, Bacteroides vulgatus, Veillonella parvula, and Bacteroides thetaiotaomicron were selected, and for propanediol pathway, Akkermansia muciniphila and Ruminococcus obeum were selected.*

### Assembly of Propionate-Producing Community

Culturing of bacteria was performed in Reinforced Clostridium Medium (Oxoid Ltd., Basingstoke, Hampshire, United Kingdom), using the Hungate tube method and under anaerobic conditions (90% N_2_/10% CO_2_). All strains were incubated at 37°C for 48 h except for *L. plantarum*, which was incubated for 24 h. At the end of the incubation, cell count was measured using flow cytometry, and standardized to 10^8^ cells ml^-1^.

Following, the consortium was subjected to the environmental conditions of the colon. Thus, the consortium culturing medium (L-SHIME medium, Prodigest NV, Zwijnaarde, Belgium) was subjected to pre-digestion simulating the passage through the upper GI tract. Gastric digestion was mimicked by maintaining pH 2 for 2 h, followed by addition of pancreatic juice [(0.9 g L^-1^ pancreatin (Sigma-Aldrich, St. Louis, MO, United States), 6 g L^-1^ Oxgall (BD, Erembodegem, Belgium), and 25 g L^-1^ NaHCO_3_ (Carl Roth GmbH, Karlsruhe, Germany)]. pH was adjusted to 6.8, and medium was incubated for 2.5 h at 37°C to simulate the small intestine digestion. pH was adjusted to 6.8 prior addition of the bacterial cocktail.

Then, 5 ml of each strain were mixed under anaerobic conditions. The consortium was prepared by transferring 5 ml of the bacterial cocktail (10% v/v) to an anaerobic glass bottle containing 45 ml of pre-digested medium. Co-culture occurred for 48 h, and 40% of the medium was replaced after 24 h. The consortium was harvested after 48 h, when the average propionate concentration was 34.5 mM (Supplementary Figure 4). The viable cell count of the consortium was 10^8^ cells ml^-1^ when administered to the M-SHIME.

### Dynamic Simulation of the Colon Environment

The mucosal simulator of the human intestinal microbial ecosystem (M-SHIME) is an *in vitro* model including both mucosal and luminal microbiota and simulating the digestive processes in the human intestinal tract (Van den Abbeele et al., 2012). We initially applied the model to evaluate the impact of a single dose of the consortium, in comparison with repeated doses, in separate SHIME runs. The experiments using single vs. repeated doses were performed using fresh fecal material from one female (27 yo), and one male donor (29 yo). Validation of the repeated dosing required fecal samples from six more donors of the same age group (30 ± 5). All donors were healthy with a normal BMI and did not use antibiotics for the last 6 months.

The M-SHIME setup consisted of double-jacketed reactors representing the stomach, small intestine and colon (Truchado et al., 2017). We simulated the environment of the transverse colon and thus, the pH was between 6.3 and 6.5, and the volume was 660 mL calculated upon retention time. Nutritional medium composition was described previously (Truchado et al., 2017), and each colon vessel had a mucosal environment consisting of 80 mucin agar-covered microcosms (AnoxKaldnes K1 carrier; AnoxKaldnes AB, Lund, Sweden), placed in a polyethylene netting (Zakkencentrale, Rotterdam, The Netherlands) (Van den Abbeele et al., 2012). Each M-SHIME vessel was inoculated with 8% (w/v) fecal slurry (Molly et al., 1993; Possemiers et al., 2004). Static incubation was completed for the first 16 h, to allow for initial stabilization of the system. After 16 h, the peristaltic pumps were started up to supply each colon vessel with 200 mL of pre-digested feed three times per day every 8 h. Pre-digestion consisted of a 45 min incubation in the stomach-small intestine compartment. All reactors were flushed with N_2_ to ensure anaerobic conditions. A scheme of the M-SHIME is presented in supplementary material (Supplementary Figure 5).

After 10 days of stabilization, 33.9 mg L^-1^ of clindamycin (Sigma-Aldrich, St. Louis, Mo, United States) were added to all colon vessels twice per day for 3 days, to trigger dysbiosis. Four days after the last antibiotic treatment, a single dose (45 ml, 6.8% of volume) of the propionate-producing consortium (PPC) was added to triplicate treatment vessels, while the other three reactors were kept as controls. Three days after the single dose, three consecutive doses of the treatment were added again for three consecutive days. The system was monitored for further 4 days to investigate the further effect of the consortium. The whole experiment ran for 27 days in case of the first 2 donors and 23 days for the six donors. Samples for VFA analysis and for DNA extraction were collected every day before the first medium replacement. Samples were collected from the M-SHIME every day and analyzed for SCFA. Samples that were sent for Illumina sequencing were samples from different days that present the end of the different phases of the SHIME run. Lumen samples were collected at days 11, 17, 20, and 23 representing stabilization phase, antibiotic treatment phase, PPC treatment phase and washout phase, respectively. The mucin samples were collected less frequently since mucin beads were changed every other day, so the samples collected were at days 9, 14, and 21 presenting stabilization phase, antibiotic treatment phase, and after PPC treatment phase, respectively.

### Single vs. Multiple Donor Experiment

In single donor experiment, the SHIME reactors were inoculated with the fecal sample of one human donor having 3 replicates for control condition and 3 replicates for treatment condition. Single donor experiments were run to ensure technical reproducibility of our experimental setup. For further validation of the results from the single donor experiments, multiple donor experiment was run using fecal inoculum from six different human donors. In the multiple donor experiment, the SHIME included 12 reactors representing colon vessels. Each 2 reactors were inoculated with a fecal sample from one single donor representing one control reactor and one treatment. Samples for SCFA and DNA analysis were collected everyday from each reactor and analyzed separately. The results from SCFA analysis and illumina sequencing were pooled together for statistical analysis.

### Community Functionality and Composition

#### SCFAs Extraction

Short-chain fatty acids were used as benchmarks of community activity, and were collected from the luminal compartment, and extracted with diethyl ether (De Weirdt et al., 2017). Total SCFA production was defined as the sum of the molar concentrations of acetate, propionate, butyrate, valerate, caproate, isobutyrate, isovalerate and isocaproate (De Weirdt et al., 2017). Differences in VFA concentrations among treatments were compared using a repeated measures mixed model, with the lsmeans adjustment and Sidak correction for multiple comparisons (GraphPad Prism 7.04, La Jolla, CA, United States). Statistical significance was assumed at *P* < 0.05.

#### DNA Extraction and Illumina Library Generation

Total DNA from luminal and mucosal samples was extracted using physical disruption with the bead beating method (Hernandez-Sanabria et al., 2010). Briefly, samples were thawed, manually homogenized, and centrifuged at 14,600 ×*g* for 5 min at 4°C. The pellet was resuspended in 1 ml of lysis buffer (100 mM Tris pH8, 100 mM Na EDTA pH8, 100 mM NaCl, 1% (w/v) polyvinylpyrrolidone, 1% PVP40, and 2% (w/v) sodium dodecyl sulfate) and transferred to a 2 ml microcentrifuge tube containing 0.3 g of zirconium beads (diameter, 0.1 mm). The cells were lysed in a Power Lyzer 24 (Mo Bio Laboratories, Carlsbad, CA, United States) for 3 min at 4800 rpm. DNA concentration and quality were verified based on the absorbance at 260 and 280 nm, using a DeNovix DS (Thermo Fisher Scientific, Waltham, MA, United States).

The V3–V4 hypervariable region of the 16S rRNA gene was amplified using primers 341F and 785R. Illumina sequencing adapters and dual-index barcodes were added to the amplicon, using a limited-cycle PCR that included an initial denaturation step at 95°C for 3 min, 15 cycles of a denaturation step at 95°C for 30 s, an annealing step at 55°C for 10 s, an extension step at 72°C for 45 s, and a final extension at 72°C for 5 min. Following, a clean-up step was performed using the AMPure XP beads (Beckman-Coulter, Krefeld, Germany) to remove free primers and primer-dimer species from amplicons. A second PCR to attach the specific Illumina multiplexing sequencing primers and index primers, was performed. Thermal cycling included an initial denaturation step at 95°C for 3 min, 8 cycles of a denaturation step at 95°C for 30 s, an annealing step at 55°C for 30 s, an extension step at 72°C for 30 s, and a final extension at 72°C for 5 min.

These PCR products were verified by gel electrophoresis, purified using the Promega Wizard PCR clean-up kit (Promega, Madison, WI, United States) following the manufacturer’s instructions and quantified with the QuantiFluor dsDNA System kit (Promega, Leiden, The Netherlands). High-throughput amplicon sequencing of the V3–V4 hypervariable region (Klindworth et al., 2013) was performed with the Illumina MiSeq platform according to the manufacturer’s guidelines at LGC Genomics GmbH (Berlin, Germany). Contigs were created by merging paired-end reads based on the Phred quality score (of both reads) heuristic as described by Kozich et al. (2013) in Mothur (Schloss et al., 2009) (v.1.33.3). Contigs were aligned to the SILVA database and filtered from those with (i) ambiguous bases, (ii) more than 10 homopolymers, and (iii) those not corresponding to the V3–V4 region.

Chimera removal and operational taxonomic unit (OTU) clustering of the sequencing reads was performed using UCHIME, with the nearest neighbor clustering algorithm implemented in mothur, at 0.03 distance (Edgar, 2010). Phylotype representatives were then generated by clustering at 97% similarity (1 mismatch), with a confidence level of at least 80 with Cyanobacteria, Eukaryota, and Archaea lineages removed. For taxonomic classification, sequence composition of the dataset was compared using the RDP Classifier tool (Wang et al., 2007), and the RDP trainset (Cole et al., 2009) version 9. Quality of the sequencing and post-processing pipeline was verified by incorporating mock samples (*n* = 12 species) in triplicate into the same sequencing run. After examining read counts, if any OTU was not classified up to genus level, the consensus sequence was blasted using the SILVA database (Pruesse et al., 2012) to obtain the taxonomic classification. In addition, all OTU sequences were aligned with those obtained from the sequencing of the 16S rRNA genes of each species, using Clustal Omega^1^.

### Community Composition and Dynamics

Data was imported into R using phyloseq (McMurdie and Holmes, 2013), and taxon abundances were rescaled by calculating the taxon proportions and multiplying them by the minimum sample size (*n* = 24789) present in the data set (McMurdie and Holmes, 2014). Alpha diversity was initially estimated within each sample using Richness, Fisher’s diversity, Shannon, Simpson, and inverse Simpson indices. Inverse Simpson was the metric used for final assessment. Pielou index was used as indicator of evenness in the community (Grunert et al., 2016). Differences in alpha diversity and evenness measures among treatments were compared using a repeated measures mixed model in SAS, using the lsmeans adjustments and Bonferroni correction for multiple comparisons (version 9.4, SAS Institute, Cary, NC, United States). To confirm these results, comparisons between control and treatment were performed using a 2-way Anova (Sidak’s method) and between and within time points using Tukey’s method in GraphPad (GraphPad Prism 7.04, La Jolla, CA, United States) (Figure 3 and Supplementary Tables 3, 4).

Beta diversity estimates based on Chao and Bray-Curtis indices were used to examine dissimilarity and determine the impact of treatment and time on microbial community structure. Principal Coordinate Analysis (PCoA) was employed to visualize the differences among samples, using the vegan package in R (Oksanen et al., 2007) (Supplementary Figure 7). Stratified permutational multivariate analysis of variance (PERMANOVA) with 999 permutations was conducted to indicate the significance of time and treatment on the microbial community differences. ANOVA was applied to reveal whether the distribution of the genera was different between treatments over time (Oksanen et al., 2007). Because of the over-dispersion in the OTU counts data, a zero-inflated count model was used to assess the effect of time and treatment and the interactions between time^∗^treatment on each individual genus. Zero-inflated models explain the excess of zeros by modeling the data as a mixture of a Poisson distribution or a negative binomial distribution. When a zero count is observed there is the zero-inflation probability, because the observation came from the always-zero distribution. When the underlying count distribution is a Poisson distribution, the model is called a zero-inflated Poisson distribution and if the count distribution is a negative binomial distribution, the mixture is called a zero-inflated negative binomial distribution. The final model was selected based on the Akaike Information Criterion (AIC). Differences among library size sample were accounted for with the offset option in proc GLIMMIX in SAS (Paschold et al., 2012). *P*-values for each comparison were converted to *q*-values that were then used to identify differences in relative abundances of bacterial genera while controlling false discovery rate (FDR) at the 5% level (Storey and Tibshirani, 2003).

Bipartite networks were inferred to highlight functional associations among bacterial genera and metabolites, using a pair-wise similarity matrix obtained from a Regularized Canonical Correlation Analysis (Lê Cao et al., 2011). Values of the similarity matrix were computed as the correlation between the relative abundances of bacterial genera and the metabolic variables, projected onto the space spanned by the first components retained in the analysis. Three relevant components were obtained setting a threshold of *r* ≥ 0.7 and genera were disseminated in the plot, in close relation with the variables correlated (De Weirdt et al., 2017).

### Enumeration of Microbial Cells Using Flow Cytometry

To assess variation in cell counts in lumen and mucin compartments, we analyzed SHIME samples collected at different time points from different phases of the SHIME run (stabilization phase, after antibiotic phase, after treatment phase and washout phase).

#### Cell Counts From Lumen and Mucin Samples

Samples used for cell counts were frozen at -20°C. All samples were diluted 1:1 in filter-sterilized PBS, and vortexed for 1 min at maximum speed. Mucin samples were disrupted for 40 s at 1800 rpm (Power Lyser 24, MO BIO Laboratories, Carlsbad, CA, United States), and centrifuged at 500 ×*g* for 4 min, while lumen samples were centrifuged at 500 ×*g* for 2 min. Supernatants were collected and passed through 20 μm filters (Filcon, BD Medimachine, Erembodegem, Belgium) to remove particulate matter. Filtered samples were then diluted 5000 times in filter-sterilized PBS, and 198 μl of the diluted sample was stained with 2 μl of SYBR Green (SG) (10,000 × diluted from stock; Invitrogen, Carlsbad, CA, United States) in 96 flat-bottom well plate. The plate with the stained samples was incubated for 20 min at 37°C. Flow cytometric analysis of the microbial cells present in the suspension was performed using a C6 plus Accuri flow cytometer (BD Biosciences, Erembodegem, Belgium) equipped with a 488 nm laser, following previously described methods (Props et al., 2017). Fluorescence events were monitored using the FL1 533/30 nm and FL3 > 670 nm optical detectors. Forward and sideways-scattered light was also collected. The BD Accuri CSampler software was used to gate and separate the microbial fluorescence events on the SSC-A and FITC-A density plot from the lumen and mucin SHIME sample background. Gating was evaluated using a 0.2 μm filter. A threshold value of 1,000 was applied on the FL1 channel. The gated fluorescence events were evaluated on the SSC-FL1 density plot, to exclude remaining background events and to obtain an accurate microbial cell count. Instrument and gating settings were kept identical for all samples.

For quantification of absolute numbers of each taxon, samples were rescaled by multiplying the relative abundance of each genus by the flow cytometry cell counts.

### Assessment of the Mitochondrial Membrane Potential

Propionate promotes flux of Cl^-^ to the mucosa, increasing the short-circuit current, hence stimulating colonic contractions through a change in potential difference (Yajima, 1988). As reduced intestinal motility and low-grade inflammation are markers of metabolic syndrome (Müller et al., 2018), we assessed the capability of the PPC to change the mitochondrial membrane potential (ΔΨ_m_). We used an *in vitro* model of the gut epithelium to reveal whether the supplementation of the consortium could potentially restore disrupted membrane potential, as observed in metabolic syndrome. Caco-2 cells were seeded onto opaque clear bottom 96-well plates (Corning, NY, United States) at a density of 20,000 cells/well and maintained for 72 h. Then, cell culture media was removed, and cells were exposed to the treatments in Table 2.

**Table 2:** Treatments added to epithelial cell model to assess changes on membrane potential.

Treatment	Concentration	Time
Clindamycin hydrochloride	33.9 mg/L	24 h
Filter-sterilized PPC (1:5 v/v in DMEM)	2:1 Acetate:Propionate ratio	24 h
CLN + PPC	33.9 mg/L + P/A/B	24 h
Cell culture medium (negative control)	–	24 h
CCCP (positive control for disrupted ΔΨ_m_)	50 μM	2 h
DMSO (control for CCCP vehicle)	<0.01%	2 h


*A negative control was used in which the cells did not get any treatment or disruption. CCCP was used as a positive control for disruption of membrane potential. The metabolites from the propionate-producing consortium (PPC) were added above the cells after being filter-sterilized. To assess the ability of the consortium (PPC) to restore the disruption caused by clindamycin (CLN), its metabolites were added together with clindamycin above the epithelial cells. P/A/B, propionate/acetate/butyrate mixture at the ratios produced by the consortium (Supplementary Figure 4).*

All the compounds were diluted from the stock solution in DMSO to the corresponding working solution in DMEM without supplementation. Filter-sterilized consortium was diluted 1:5 (v/v) in DMEM without supplementation. DMSO was used as a control.

After exposing the cells to the treatments for 24 h at 37°C (95% humidity, 10% CO_2_)_,_ cells were washed once with 200 μl of PBS with Ca^++^ and Mg^++^ (Sigma) and 100 μl of JC-1 stain (10 μM in DMEM) (Cayman Chemical, MI, United States) were added to the wells and incubated for 20 min. Following, wells were washed with PBS supplied with Ca^++^ and Mg^++^ and 10% FBS, and 100 μl of DMEM were added to each well before measuring with a SpectraMax Plus Microplate Reader (Molecular Devices, LLC). Excitation/emission wavelength pairs were set at 475/530 nm and 475/590 nm, for JC-1 monomer and aggregate detection, respectively. The background (A590 of non-stained cells) was subtracted from the test signals. Results were expressed as the ratio between aggregate/monomer. All Pairwise Multiple Comparison Procedures and Statistical analysis was done using Holm-Sidak method in GraphPad (GraphPad Prism 7.04, La Jolla, CA, United States).

## Results

We assessed the reproducibility of the simulated clindamycin-induced dysbiosis using the Mucosal Simulator of the Human Intestinal Ecosystem (M-SHIME), before evaluating the potential of the PPC to restore functionality. Two M-SHIME runs were separately conducted with fecal microbiota from two volunteers, and treatments were supplied to triplicate reactors. Concentrations and profiles of SCFA in the luminal content of the proximal colon compartments across the three technical replicate reactors were found to be reproducible (Supplementary Figures 1, 2).

### Gut Microbiome Functionality Is Improved When a Propionate-Producing Consortium Is Supplied After Antibiotic Use in Donor 1

Altered production of SCFA is considered one of the hallmarks of dysbiosis, and thus, we validated that clindamycin disrupted the fermentation pattern of the simulated gut ecosystem. Upon antibiotic supplementation, propionate and butyrate decreased by approximately 57 and 95%, respectively, and remained consistently and significantly low (*P* < 0.05) across all triplicate experiments for both donors 1 and 2 (Supplementary Figures 1, 2). In the single donor experiments, we found that one single dose of the PPC did not promote functional recovery on either of the donors (Supplementary Figures 1, 2), whereas three consecutive doses of the consortium triggered a significant increase in propionate production only in donor 1 (14.84 ± 1.06 mM; *P* < 0.05, Supplementary Figure 1). This positive outcome remained consistent across the three replicates until the end of the experiment (Supplementary Figure 1 and Supplementary Table 1). As the impact of one single dose of the consortium seemed to be marginal, we decided to use three consecutive doses in a multiple-donor experiment. We conducted an extra M-SHIME run with fecal samples from six different donors of the same age group (30 ± 5 yo). Functional recovery and inter-individual variability were assessed, and all the following results presented were from the multiple-donor experiment.

### Modulation of Microbiota Functionality by a Propionate-Producing Consortium Upon Antibiotic Induced Dysbiosis

**FIGURE 1:**
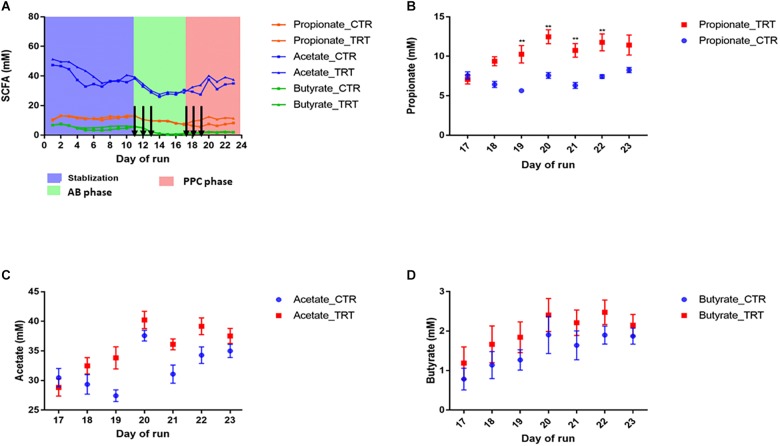
Addition of the propionate-producing consortium (PPC) promotes recovery of propionate production after antibiotic-associated disruption. CTR: Control, TRT: Treatment, AB: Antibiotic. **(A)** Short chain fatty acid production during the three different phases of the experiment: Stabilization, post-antibiotic disruption, and post-addition of the propionate-producing consortium. Antibiotic treatment was added on days 11, 12, and 13. Treatment was added on days 17, 18, and 19. Days in which antibiotics and PPC were added were indicated by arrows. **(B)** Propionate was the main short chain fatty acid impacted after 2 doses, as propionate levels significantly increased from day 19 in the treatment reactors (*P* < 0.05). No significant difference was detected for acetate **(C)** and butyrate **(D)** after the treatment was added. ^∗∗^*P* < 0.001.

After disruption with clindamycin, there was a significant and consistent decrease in butyrate, propionate, and acetate by 88, 46, and 16%, respectively (*P* < 0.05) (Figure 1). Inter-individual differences in SCFA production were observed upon dosing the PPC. Repeated supplementation of the consortium promoted significant increase in propionate production (12.47 ± 0.88 mM; *P* < 0.05), compared with the control (7.57 ± 0.37 mM), resulting in nearly 100% recovery of the initial propionate concentrations. Restoration of butyrate and acetate was variable across the six different donors and no significant recovery was observed (Figure 1). These observations confirm that our designed microbial consortium led to effective functional recovery of propionate production following dysbiosis.

### Propionate-Producing Consortium Supports the Partial Recovery of the Epithelial Mitochondrial Membrane Potential After Antibiotic Disruption

**FIGURE 2:**
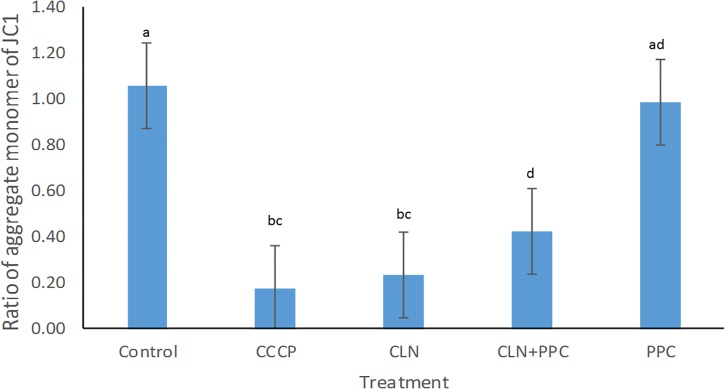
Propionate-producing consortium triggered a partial recovery for the membrane potential after clindamycin disruption. Following clindamycin (CLN) supplementation, membrane potential was significantly higher when one dose of the propionate-producing consortium (PPC) was provided, in comparison with exposure to clindamycin alone (*P* < 0.05). Protonophore carbonyl cyanide m-chlorophenylhydrazone (CCCP) was used as a negative control to disrupt membrane potential. All Pairwise Multiple Comparisons between treatments were assessed using Holm-Sidak method with overall significance level equal to 0.05. Significant differences were indicated using different superscripts. Presence of the same letter in the superscript indicates that samples were not significantly different (*P* > 0.05).

Mitochondrial membrane potential of epithelial cells is fundamental for gut motility as well as for cell division, as it modulates the distribution of several conserved cell division proteins (Strahl and Hamoen, 2010). Carbonyl cyanide m-chlorophenylhydrazone (CCCP) was used as a positive control for the disruption of the membrane potential, because it rapidly disperses the proton motive force (pmf) or membrane potential (Strahl and Hamoen, 2010). A significant decrease in membrane potential was observed when CCCP without consortium was added to our cell model, confirming the negative effect of the CCCP on the membrane potential (Supplementary Table 2, *P* < 0.05, Figure 2). Clindamycin decreased the membrane potential by approximately 80% (*P* < 0.05), confirming the negative effect of the antibiotic toward epithelial cells. When clindamycin was added together with the PPC, the ratio of the aggregate monomer of the JC1 was increased by 40% (*P* < 0.05), indicating partial recovery of the membrane potential after clindamycin disruption.

### Antibiotic Use Significantly Decreased the Bacterial Cell Load

A workflow for the quantitative microbiome profiling can be built through parallelization of amplicon sequencing and flow cytometric enumeration of microbial cells (Vandeputte et al., 2017). Flow cytometry revealed a significant decrease in the cell count after antibiotic use in both lumen and mucin compartments (*P* < 0.05). Administration of the PPC did not impact the total cell load (*P* > 0.05*)*. However, cell count decreased in the reactors that were not supplemented (Supplementary Figure 6).

### Propionate-Producing Consortium Shaped the Bacterial Community Based on Number of Doses and Host Influence

**FIGURE 3:**
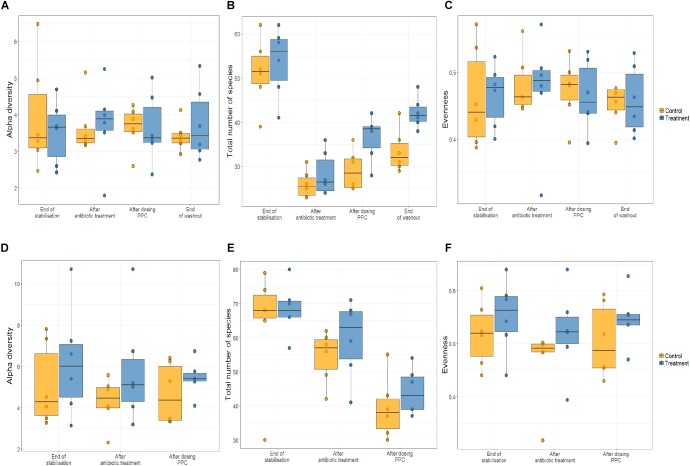
Propionate-producing consortium triggered an increase of total number of species after the washout period only in the lumen compartment. Relative abundance was quantified at the end of stabilization phase, antibiotic treatment phase, propionate-producing consortium phase, and washout phase. No effect of the consortium on the alpha diversity of lumen **(A)** and the mucin **(D)** were detected. Antibiotic supplementation triggered significant decrease in the total number of species (*P* < 0.05) only in the lumen compartment **(B)**, but not in mucin compartment **(E)**. The propionate-producing consortium significantly increased the total number of species at the end of the washout **(B)**. No effect of the consortium on the evenness of the community in the lumen **(C)** and the mucin **(F)** was observed.

As shifts in bacterial taxa and decrease in community diversity are benchmarks of dysbiosis, we dynamically monitored both luminal and mucosal communities using the M-SHIME. Alpha diversity was unchanged in control and treatment reactors in the multi-donor experiment upon consortium supplementation (Figure 3A).

**FIGURE 4:**
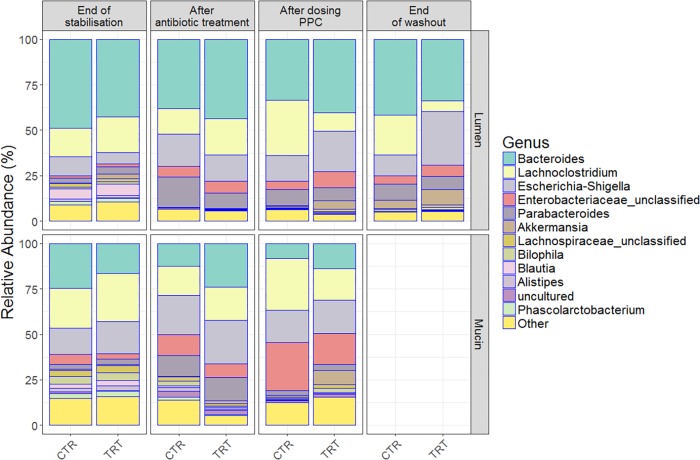
Relative bacterial abundances on the luminal and mucosal compartments shifted among end of stabilization, after antibiotic use, after 3 doses of PPC and after washout. Genera with the highest relative abundances across time points were uncovered in the **(A)** lumen compartment and **(B)** mucosal compartment. Six replicates (donors) were averaged to highlight the inter-individual effect of the propionate-producing consortium.

Clindamycin significantly decreased richness in all luminal compartments (Figure 3B and Supplementary Table 3), but consecutive doses of the PPC triggered a significant recovery of this metric in the lumen compartment (*P* < 0.05). The difference in richness between the control and treatment was not significant until the end of the washout period, when the treatment reactors had significantly higher total number of species than the control reactors (*P* < 0.05, Figure 3B and Supplementary Table 3). Total number of species tended to be higher in the mucin compartment as well. As the mucosal communities are in close contact with the host, successful function transfer in the mucosal compartment may be relevant for possible host effects (Figure 3E and Supplementary Table 4). In addition, evenness of mucosal communities following antibiotic-induced dysbiosis tended to be lower in comparison with that at the end of the stabilization period (Figure 3F and Supplementary Table 4). In contrast, evenness in the lumen community after dysbiosis remained constant, suggesting that mucus bacteria are more sensitive to antibiotics than bacteria in the lumen environment (Figure 3C and Supplementary Table 3). We observed that antibiotic supplementation promoted both loss of bacterial richness and increased relative abundance of Enterobacteriaceae and *Escherichia-Shigella*, albeit not significant for the latter in the mucin (Figure 4 and Supplementary Table 5D). Importantly, relative abundance of *Escherichia*-*Shigella* was significantly decreased in the lumen when the consortium was supplied (Supplementary Table 5C). Pathologies characterized by microbial dysbiosis have in common a decrease in the community composition complexity, as well as an increase in aero-tolerant genera such as Enterobacteriaceae (Vonaesch et al., 2018). Although microbial community can be deeply disrupted upon antibiotic use, our results indicate that the PPC shaped the community based on the number of treatment doses and donor.

Repeated doses of the consortium confirmed a potential recovery of the community, both through direct engraftment and through indirect reinforcement of other propionate producers. For instance, unclassified Lachnospiraceae, *Akkermansia* and *Bacteroides* increased their relative abundance in both compartments, indicating that some members of our consortium (*Coprococcus catus*, *Akkermansia muciniphila*, *Bacteroides* *thetaiotaomicron*, and *B*. *vulgatus*) may have been successfully engrafted in the mucin compartment (Figure 4 and Supplementary Table 5B). Moreover, the presence of these genera even after the washout may suggest that indirect positive reinforcement of the overall community may have ensued. Long-lasting increased relative abundance of *Akkermansia* species seem to be consistent across individuals, as indicated by the community composition at the end of the washout period (Figure 4). Moreover, the relative abundance of other species, such as *Veillonella* (Supplementary Table 5A), indicate that the intra-individual engraftment and indirect reinforcement of the consortium may be enduring.

### Addition of the Propionate-Producing Consortium Amends Community Metabolic Networks Following Environmental Disruption

Relevance networks analyses indicated that SCFA-production networks shifted with antibiotic treatment. As metabolic products were significantly decreased when the environmental disruption happened, larger networks including bacteria associated with acetate production were observed (Supplementary Figure 3A). Increased acetate concentrations seemed to correlate with increased relative abundances of *Bifidobacterium*, *Faecalibacterium*, *Flavonifractor*, and *Anaerotruncus* (Supplementary Figure 3A and Supplementary Table 5C). With respect to butyrate, a positive association was only found with increased relative abundance of *Bacteroides.* No genera were significantly associated with high concentrations of propionate, after antibiotic was provided (Supplementary Figure 3A) (*P* < 0.05).

**FIGURE 5:**
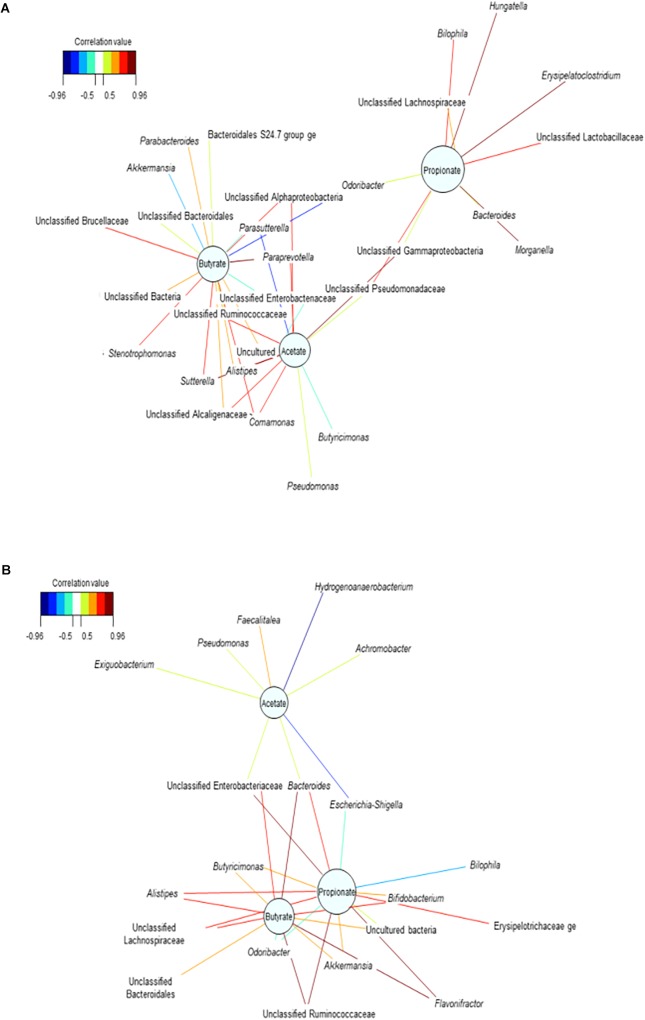
Bacterial interactions networks influenced the production of the major short chain fatty acids over time. **(A)** Treatment reactors at day 20 after adding 3 doses of the propionate consortium, **(B)** Control vessels at day 20. These bipartite networks are based on the regularized canonical correlations between relative bacterial abundances and relative concentrations of the main SCFA. Interactions have been filtered for an absolute correlation above 0.8 and are colored following the key shown. Significant interactions are shorter lines, and genera with similar abundances within SHIME compartment tend to cluster closely.

The relevance network for the control reactors displayed intermingled propionate and butyrate networks (Figure 5B). This may suggest that the community competes for the substrate available, preventing from significantly increasing propionate production. Upon repeated dosage of the consortium, the propionate network showed that higher relative abundances of Unclassified Lactobacillaceae, *Morganella*, *Hungatella*, *Erysipelatoclostridium*, Unclassified Lachnospiraceae and *Bilophila* were associated with higher concentrations of propionate (Figure 5A and Supplementary Table 5C). The increased relative abundance of these and other genera such as *Parabacteroides* in the mucin (succinate producer) may confirm the indirect positive reinforcement of the PPC on the overall community (Supplementary Table 5C). Moreover, *Hungatella* has been reported to thrive on medium used to produce probiotic bacteria (Kaur et al., 2013).

Finally, the enduring indirect impact of the consortium was validated on the propionate network at the end of the washout period (Supplementary Figure 3B). We observed that higher relative abundance of Unclassified Lactobacillaceae was positively associated with increased concentrations of propionate (*P* < 0.05), potentially revealing that acetate-producing bacteria belonging to this family (*Lactobacillus* sp.) actively participate in the functional recovery of the community. The lasting effect of the consortium may as well indicate the successful engraftment of one of the genera comprised in this community. Higher abundances of *Akkermansia* were associated with higher concentrations of acetate at the end of the washout (Supplementary Figure 3B). This could be an additional indicator of the successful adaptation and operational efficacy of the consortium.

### Variations in Cell Densities Confirmed the Impact of the Propionate-Producing Consortium in the Compositional Dynamics of the Community

**FIGURE 6:**
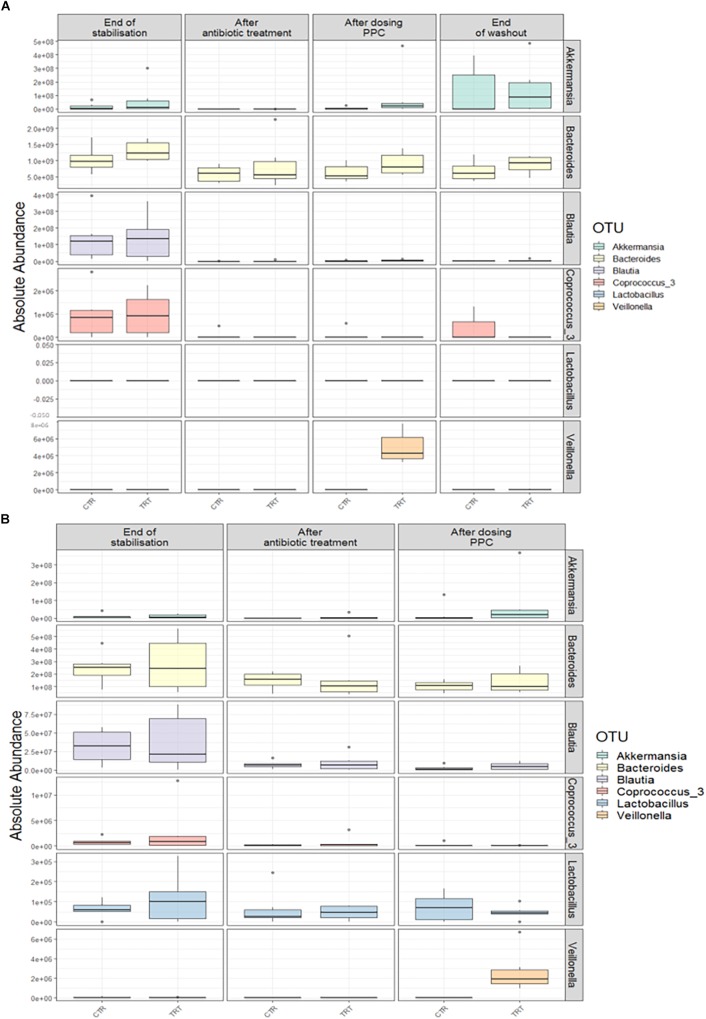
Absolute abundance of the seven genera included in the propionate-producing consortium. Quantification of absolute abundances revealed the direct engraftment of specific genera in the lumen **(A)** and mucin **(B)** compartments. *Veillonella* showed a significant increase (*P* < 0.05) in both lumen and mucin compartments after the administration of the propionate-producing consortium.

**FIGURE 7:**
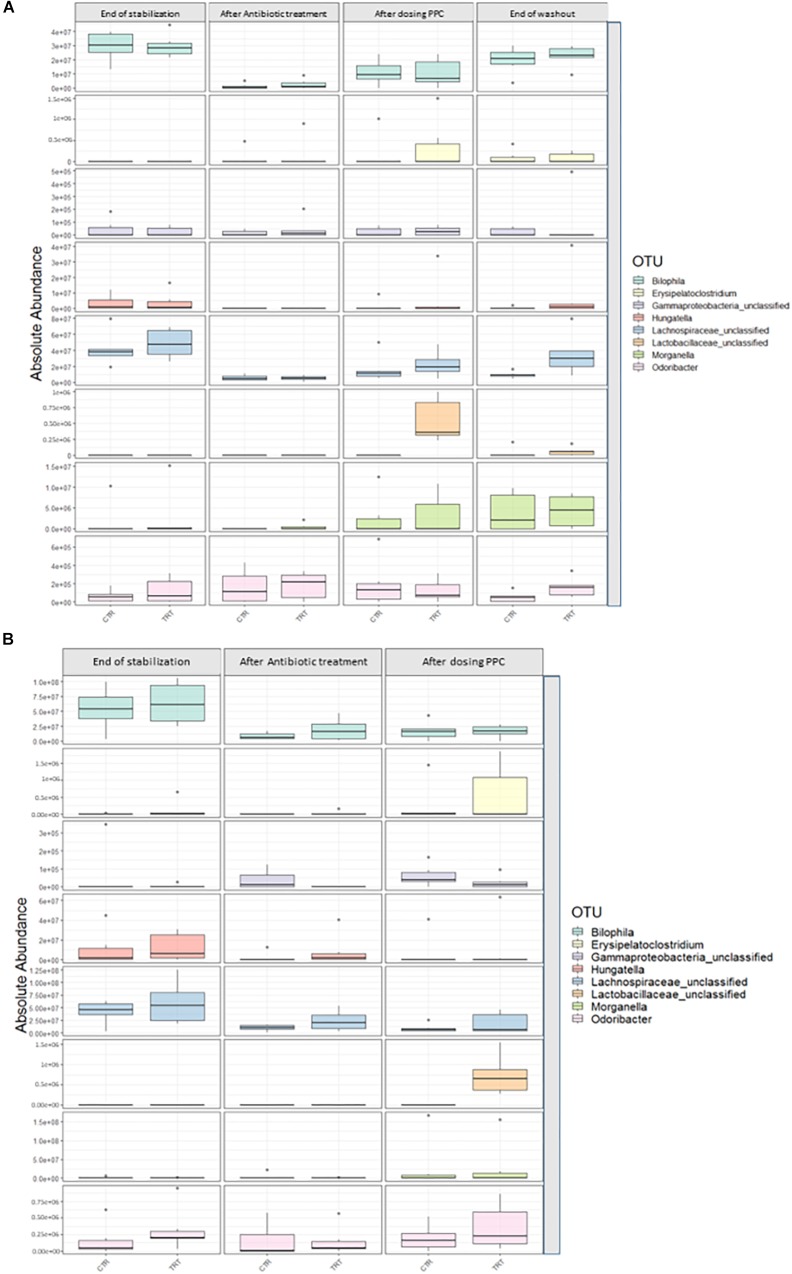
Absolute abundance of genera involved propionate production upon consortium supplementation. Quantification of absolute abundance validated the indirect reinforcement of specific genera in the lumen **(A)** and mucin **(B)** compartments. Lactobacillaceae was significantly increased in both lumen and mucin compartments (*P* < 0.05) upon administration of the propionate-producing consortium. Lachnospiraceae showed a significant increase only in the lumen compartment at the end of the washout phase (*P* < 0.05).

We performed absolute quantification of the taxa detected in our study, to comprehensively explain the differences observed in the relative abundances. Our results confirmed the direct engraftment of one of the members of our consortium, as the absolute abundance of *Veillonella* significantly increased in both luminal and mucosal compartments of the treatment reactors after providing 3 doses of the consortium (*P* < 0.05) (Figure 6 and Supplementary Tables 6, 7). As for the indirect reinforcement, relevance networks initially suggested that some genera were linked to propionate production. We observed a significant increase in absolute abundance of unclassified Lactobacillaceae after adding the 3 doses of the consortium in both lumen and mucin (*P* < 0.05) (Figure 7 and Supplementary Tables 8, 9). Moreover, unclassified Lachnospiraceae significantly increased in the treatment vessels after the washout period in the lumen compartment (*P* < 0.05) (Figure 7 and Supplementary Table 8). Although *Bilophila* showed increased relative abundance after adding the 3 doses of the consortium, absolute abundance was not increased (Figure 7 and Supplementary Tables 8, 9). The combined approach of relative and absolute abundances assisted to elucidate the course of action of the PPC. Thus, the observed positive effect of the consortium may be explained as a synergic impact on the cell counts and on the relative abundances of taxa involved in propionate production pathways.

## Discussion

In our study, we aimed at engineering our PPC considering the three major pathways for propionate production (acrylate, succinate, and propanediol pathways) (Reichardt et al., 2014). The consortium positively impacted functionality and composition of the microbial community and supported the partial recovery of membrane potential after clindamycin disruption.

SCFA analysis from the luminal SHIME samples revealed a significant drop in bacterial metabolic activity after clindamycin-induced dysbiosis. Previous studies reported the ability of antibiotics to significantly influence taxonomic richness, diversity and evenness (Dethlefsen et al., 2008; Dethlefsen and Relman, 2011; Francino, 2016; Rojo et al., 2017), as observed in the lumen and mucosal communities of our experiment. In addition, flow cytometry analysis confirmed the negative the effect of dysbiosis on bacterial cell counts, as they significantly decreased upon antibiotic administration. In the single donor experiment, one single dose of the PPC did not impact neither functionality measured by SCFA production nor microbial community. The dose we provided may have not been enough for a beneficial effect, as in the case of probiotic strategies that can only confer a positive effect when administered in adequate amounts (Ouwehand, 2017). Repeated doses of our consortium showed a significant effect on the functionality in one of the 2 donors of the single-donor experiments, and in all six samples from six individual donors in the multiple-donor experiment, with full recovery of propionate production. Resident bacteria potentially interacted with the supplied PPC, as those administered may have provided metabolites like acetate, lactate, and propionate. Thus, resident bacteria cross-feeding on these metabolites eventually led to the production of propionate (Derrien and van Hylckama Vlieg, 2015). Our results suggest that restoration of propionate production by our engineered consortium was successful.

Despite the positive impact of the PPC, some elements of dysbiosis remained unchanged. For instance, the consistent relative abundance of Proteobacteria like *Escherichia-Shigella* and *Bilophila* before and after treatment, indicates that those bacteria can act as opportunistic pathobionts in cases of dysbiosis. *Bilophila* may have utilized mucin-degradation products from our consortium to produce propionate. The positive correlation between *Bilophila* and propionate may be an indicator of the inflammatory status of the environment (Feng et al., 2017). However, absolute abundance of this genus was not increased when the consortium was provided. Instead, members from our PPC like *Bacteroides* and *Akkermansia* may have occupied the niche of primary carbohydrate degradation and subsequently promoted fermentation by resident bacteria, as is the case for mucin metabolism. Mucin degradation can liberate sugars, amino acids, sialic acids, and sulfate that can be consumed as substrates by the resident commensals (Derrien and van Hylckama Vlieg, 2015) or even by the provided bacteria. Our consortium contained a strain of *Akkermansia muciniphila*, a specialized mucin-degrading bacterium (Chia et al., 2018), which could have provided sugar monomers from mucin upon degradation. Mucin-derived sugars like fucose could be utilized by *Akkermansia muciniphila* (Ottman et al., 2017) or by *Ruminococcus obeum* (Lachnospiraceae) to produce propionate through the propanediol pathway (Flint et al., 2014; Reichardt et al., 2014). This could explain the increase in the relative abundance in *Akkermansia* and Lachnospiraceae after administering the three doses of our consortium.

Ingested bacteria can impact resident communities through at least three different mechanisms: through trophic interactions, a direct alteration in fitness, or an indirect alteration in fitness through altered production of host-derived molecules (Derrien and van Hylckama Vlieg, 2015). One of the markers considered for successful colonization from bio-therapeutics is engraftment (Smillie et al., 2018). Engraftment originally refers to “incorporation of grafted tissue into the body of the host” (Miller et al., 2005), and it has been applied to explain the stable establishment of a bacterial strain in the human gut (Maldonado-Gómez et al., 2016). In our SHIME model, the complexity of the simulated colonic ecosystem allowed for analysing the impact of the administered bacterial community on microbial interaction networks, independently of host inputs. To ensure engraftment, long-term persistence of the different species of the live microbes in the consortium should be monitored in different donors, as described by Maldonado-Gómez et al. (2016). The beads coated with mucin in our model provided a comprehensive overview of the bacterial colonization process. For instance, we observed an increase in the relative abundance of unclassified Lachnospiraceae, *Akkermansia*, and *Bacteroides* in the mucosal compartment, after repeated doses. This indicated that some members of the consortium (*Akkermansia muciniphila*, *Coprococcus catus, Ruminococcus obeum*, and *Bacteroides* *thetaiotaomicron* and *Bacteroides vulgatus*) may have been successfully engrafted in the mucin compartment. In our experiment, we attained long-lasting increased relative abundance of *Akkermansia* species across individuals, as indicated by the community composition at the end of the washout period. Previous reports suggest that species traits such as functionality are major drivers of bacterial colonization (Smillie et al., 2018). In this way, the functional redundancy of our consortium may have ensured prevalence in the lumen following repeated dosage, even after 4 days. *Akkermansia* is considered a common member of the autochthonous human gut microbiome, which may guarantee permanent colonization as opposed to commercial probiotics belonging to lactic-acid producing bacteria (Maldonado-Gómez et al., 2016). Nevertheless, whether higher engraftment success is a general attribute of autochthonous members of the microbiome, or whether it is specific for certain probiotic strains needs to be elucidated (Maldonado-Gómez et al., 2016).

Metabolic syndrome leads to excess cellular oxidative stress and oxidative damage of mitochondrial components, impacting mitochondrial membrane potential (Nicolson, 2007). Clindamycin is a chlorinated analog of lincomycin and inhibits basal epithelial transport (Goldhill et al., 1996), impacting electrical field stimulation (EFS) (Goldhill et al., 1996) and mitochondrial membrane potential (Goldhill et al., 1996). In our epithelial cell model, the presence of a Cl^-^ in the molecule of the drug reduced the basal short circuit current (Goldhill et al., 1996) and disrupted the mitochondrial membrane potential upon exposure to clindamycin. Proper mitochondrial membrane potential is a requirement for oxidative phosphorylation (Mitchell, 1966; Nicolson, 2007) and impaired mitochondrial oxidative phosphorylation contributes to the development of the metabolic syndrome (Ren et al., 2010). Hence, the partial recovery of the membrane potential following the addition of the PPC suggests that application of our functional community may be a promising strategy to amend microbial dysbiosis and confer beneficial effects toward host epithelium.

Nowadays, next-generation probiotics and live biotherapeutics are being developed based on core members of the microbiome (Olle, 2013), as in the case of our PPC. Endogenous core bacterial strains included in these biotherapeutics may have higher ecological fitness when administered to humans compared to the exogenous strains, such as commercial probiotics. However, the concept of ecological performance related to probiotic functionality is yet to be elucidated (Maldonado-Gómez et al., 2016). In conclusion, members of our gut microbiome can be used as new generation probiotics for targeting different health aspects. We confirmed that the established PPC can impact functionality by restoring the propionate production after antibiotic-induced dysbiosis. The key question would be if the PPC can impact beyond the ecology of the gut microbiome and influence host health. Importantly, as such consortia are to be administered orally, developing carrier matrices to ensure survival to the harsh conditions of the upper GI tract should be earnestly considered. Further research to determine dose-response outcome and long-term benefits will foster our knowledge on novel probiotic consortia. Indeed, understanding strain selection and the metabolic pathways for producing different SCFA will aid in the development of functional consortia targeted for prevention and management of major health concerns, such as metabolic syndrome, or even for personalized nutrition strategies.

## Author Contributions

RE, EH-S, and TV conceived and designed the experiments. RE and EH-S developed the methodology, and prepared the figures and tables. RE performed the reactor work and laboratory analyses. MC performed the cell work. RP contributed to the acquisition, fingerprinting, and mining of FCM data. EH-S performed the statistical analysis and interpretation of the sequencing data. TV acquired funding. RE, EH-S, MC, RP, and TV wrote and reviewed the manuscript.

## Conflict of Interest Statement

The authors declare that the research was conducted in the absence of any commercial or financial relationships that could be construed as a potential conflict of interest.

## Footnotes

1 http://www.ebi.ac.uk/Tools/msa/clustalo/
