# Supplementary material for: Propionate-Producing Consortium Restores Antibiotic-Induced Dysbiosis in a Dynamic in vitro Model of the Human Intestinal Microbial Ecosystem
Source: Front Microbiol. 2019 May 31;10:1206. doi: 10.3389/fmicb.2019.01206 (PMC6554338; doi:10.3389/fmicb.2019.01206)
Supplement: Supplementary file 1 [file Data_Sheet_1.pdf]

Supplementary Material

Propionate-producing consortium restores antibiotic-induced dysbiosis *in vitro*

Racha El Hage[1], Emma Hernandez-Sanabria[1], Marta Calatayud Arroyo[1], Ruben Props[1] and Tom Van de Wiele[1]*

[1] Center for Microbial Ecology and Technology (CMET), Ghent University, Coupure Links 653, 9000 Ghent, Belgium.

*Correspondence

Dr. Tom Van de Wiele

tom.vandewiele@ugent.be

Supplementary Table 1: Differences in major short chain fatty acid concentrations between control and treatment in the lumen at different time points (TPT) in Donor 1. **Time point (TPT) is expressed in days. Days selected were day 9 (before antibiotic treatment), day 17 (after antibiotics), day 18 (after single dose of propionate producing consortium), day 24 (after 3 doses of propionate producing treatment) and day 27 (end of the washout period).**

**SCFA**	**TPT**	**Condition**		***P* value**
		CTR (Mean % ± SEM)	TRT(Mean % ± SEM)	
Acetate	9	30.65 ± 5.32	32.59 ± 2.80	
	17	30.08 ± 1.04	31.24 ± 2.20	
	18	29.88 ± 1.20	31.25 ± 0.99	NS
	24	34.08 ± 2.95	35.69 ± 1.76	
	27	31.90 ± 2.94	34.3 ± 1.74	
Propionate	9	12.87 ± 1.38	15.09 ± 0.22	
	17	5.10 ± 0.35	5.75 ± 0.15	NS
	18	4.81 ± 0.13	5.85 ± 0.12	
	24	9.62 ± 1.58	14.84 ± 1.06	0,0059
	27	10.14 ± 1.44	14.44 ±0.89	0,0483
Butyrate	9	9.93 ± 2.46	6.40 ± 1.27	
	17	0.33 ± 0.12	0.24 ± 0.05	
	18	0.67 ± 0.09	0.43 ± 0.03	NS
	24	2.52 ± 0.23	2.48 ± 0.22	
	27	2.29 ± 0.11	2.82 ± 0.32	

Supplementary Table 2: Ratios of the aggregate monomer of the JC1 values (indicative for membrane potential) after the different treatments applied on the epithelial cells. Overall significance level = 0.05. Clindamycin treatment (CLN) showed a similar effect to CCCP which is the positive control for mitochondrial membrane potential disruption. Propionate producing consortium (PPC) showed a significant effect on membrane potential when added together with CLN, showing significant difference ($P<0.05$) with CLN treatment alone. Significant differences were indicated by different superscripts.

**Treatment**	**Mean% ± SEM**
Control	0.793 ± 0.035^{a}
CCCP	0.189 ± 0.008^{bc}
CLN	0.205 ± 0.011^{bc}
CLN+PPC	0.461 ± 0.086^{d}
PPC	0.628 ± 0.083^{ad}

Supplementary Table 3: Community structure indices in the lumen compartment. 1: End of Stabilization phase, 2: After Antibiotic use, 3: After 3 doses of propionate-producing consortium, and 4: washout phase. Inverse Simpson indicates Alpha diversity, Pielou indicates Evenness, and Total Species indicates the total number of Species or richness. Comparisons between control (CTR) and Treatment (TRT) at the same time point were performed using 2-way Anova test (Sidak's method). Differences between the same treatment at different time points were assessed using 2-way Anova (Tukey's method), and superscripts indicate significance (*P<0.05)*.

Index	TPT	Condition		*P value*
Inverse Simpson		CTR (Mean % ± SEM)	TRT(Mean % ± SEM)	
	1	3.94 ± 0.61^{a}	3.52 ± 0.36^{b}	NS
	2	3.65 ± 0.31^{a}	3.74 ± 0.46^{b}	NS
	3	3.65 ± 0.24^{a}	3.63 ± 0.39^{b}	NS
	4	3.41 ± 0.16^{a}	3.76 ± 0.41^{b}	NS
Pielou	1	0.46 ± 0.03^{a}	0.46 ± 0.02^{b}	NS
	2	0.48 ± 0.02^{a}	0.47 ± 0.04^{b}	NS
	3	0.47 ± 0.02^{a}	0.46 ± 0.02^{b}	NS
	4	0.45 ± 0.01^{a}	0.46 ± 0.02^{b}	NS
Total species	1	51.33 ± 3.16^{a}	53.67 ± 3.21^{d}	NS
	2	26.00 ± 1.26^{b}	28.33 ± 2.04^{e}	NS
	3	29.17 ± 1.85^{bc}	36.50 ± 2.08^{f}	NS
	4	33.50 ± 1.98^{bc}	42.17 ± 1.42^{f}	0.0352

Supplementary Table 4: Community structure indices in the mucin compartment. 1: End of Stabilization phase, 2: After Antibiotic use, and 3: After 3 doses of propionate-producing consortium. Inverse Simpson indicates Alpha diversity, Pielou indicates Evenness, and Total Species indicates the total number of Species or richness. Comparisons between control (CTR) and Treatment (TRT) at the same time point were performed using 2-way Anova test (Sidak's method). Differences between the same treatment at different time points were assessed using 2-way Anova (Tukey's method), and superscripts indicate significance (*P<0.05)*.

Index	TPT	Condition		*P value*
Inverse Simpson		CTR (Mean % ± SEM)	TRT(Mean % ± SEM)	
	1	5.09 ± 0.81^{a}	6.22 ± 1.09^{b}	NS
	2	4.31 ± 0.47^{a}	5.82 ± 1.09^{b}	NS
	3	4.69 ± 0.60^{a}	5.45 ± 0.35^{b}	NS
Pielou	1	0.52 ± 0.03^{a}	0.55 ± 0.03^{b}	NS
	2	0.46 ± 0.03^{a}	0.52 ± 0.03^{b}	NS
	3	0.50 ± 0.03^{a}	0.55 ± 0.02^{b}	NS
Total species	1	64.00 ± 7.10^{a}	68.33 ± 3.08^{c}	NS
	2	54.50 ± 3.10^{a}	59.67 ± 4.69^{c}	NS
	3	39.33 ± 3.68^{b}	44.17 ± 2.79^{d}	NS

Supplementary Table 5A: Bacterial relative abundances of taxa belonging to the Propionate-producing consortium (PPC) in the luminal compartment of the M-SHIME at the end of the stabilisation phase (time point 1), after clindamycin supplementation (time point 2), after 3 days of PPC administration (time point 3) and after 4 days of washout (time point 4). NS= not significantly different. Different superscripts indicate significantly different means.

Taxon	Time point	Treatment (Mean ± SEM)		Effect		
		PPC	Control	Time	Treatment	Time*Treatment
Akkermansia	1	0.0045 ± 0.002^{ab}	0.0018 ± 0.0007^{a}	0.001	0.07	NS
	2	0.0021 ± 0.009^{a}	0.00008 ± 0.00003^{b}			
	3	0.0065± 0.004^{ab}	0.0018 ± 0.0006^{b}			
	4	0.0123 ± 0.005^{b}	0.0071 ± 0.0044^{c}			
Bacteroides	1	0.0153 ± 0.001^{a}	0.0143 ± 0.002^{a}	0.03	NS	NS
	2	0.0136 ± 0.0651^{a}	0.0134 ± 0.001^{a}			
	3	0.0125 ± 0.001^{b}	0.0104 ± 0.0009^{b}			
	4	0.0126 ± 0.001^{a}	0.0112 ± 0.002^{b}			
Blautia	1	0.0012 ± 0.005^{a}	0.0014 ± 0.0006^{a}	<0.0001	NS	NS
	2	0.0008 ± 0.0003^{b}	0.0003 ± 0.0001^{a}			
	3	0.0008 ± 0.0002^{b}	0.0003 ± 0.0001^{a}			
	4	0.0021 ± 0.0007^{c}	0.0011 ± 0.0004^{a}			
Coprococcus	1	0.00001 ± 4E-6^{a}	0.00001 ± 5.4E-6^{a}	<0.0001	0.0005	<0.0001
	2	2.6E-8 ± 0.0106^{b}	3.1E-6 ± 2.86E-6^{a}			
	3	2.2E-8 ± 9.0E-9^{c}	1.9E-6 ± 1.8E-6^{b}			
	4	1.2E-7 ± 4.8E-8^{d}	6.9E-6 ± 3.9E-6^{a}			
Lactobacillus	1	Below detection limit	Below detection limit	-	-	-
	2					
	3					
	4					
Veillonella	1	0.00029 ± 0.0003^{a}	0.00019 ± 0.0002^{a}	<0.0001	<0.0001	<0.0001
	2	0.00091 ± 0.0008^{b}	0.0044 ± 0.0004^{b}			
	3	0.0074 ± 0.0007^{c}	0.0043 ± 0.0039^{c}			
	4	0.017 ± 0.012^{d}	0.0028 ± 0.0025^{d}			

Supplementary Table 5B: Bacterial relative abundances of taxa belonging to the Propionate-producing consortium (PPC) in the mucosal compartment of the M-SHIME at the end of the stabilisation phase (time point 1), after clindamycin supplementation (time point 2), after 3 days of PPC administration (time point 3). NS= not significantly different. Different superscripts indicate significantly different means.

Taxon	Time point	Treatment (Mean ± SEM)		Effect		
		PPC	Control	Time	Treatment	Time*Treatment
	1	0.0156 ± 0.005[a]	0.0078 ± 0.0003[a]			
Akkermansia	2	0.0141 ± 0.009[a]	0.0036 ± 0.0001[b]	0.001	0.09	NS
	3	0.051± 0.004[b]	0.0023 ± 0.0001[b]			
	1	0.0060 ± 0.0014	0.0064 ± 0.0014			
Bacteroides	2	0.0055 ± 0.0018	0.0049 ± 0.0007	0.06	NS	NS
	3	0.0039 ± 0.0011	0.0032 ± 0.0006			
	1	0.0007 ± 0.0002[a]	0.0007 ± 0.0002[a]			
Blautia	2	0.0003 ± 0.001[b]	0.0002 ± 0.00008 [b]	<0.0001	NS	NS
	3	0.0003 ± 0.00007[b]	0.0002 ± 0.00006[b]			
	1	0.00004 ± 3E-6[a]	0.00002 ± 5.8E-6[a]			
Coprococcus	2	0.00003 ± 0.00002[a]	4.7E-6 ± 2.01E-6[b]	0.008	NS	NS
	3	3.66E-6 ± 1.3E-6[b]	4.6E-6 ± 5.5E-6[ab]			
	1					
Lactobacillus	2	Below detection limit	Below detection limit	-	-	-
	3					
	1	0.00048 ± 0.0007[a]	0.00031 ± 0.0004[a]			
Veillonella	2	0.000015 ± 0.00002[b]	0.00015 ± 0.00002[b]	<0.0001	NS	NS
	3	0.0076 ± 0.0001[c]	0.0013 ± 0.0019[c]			

Supplementary Table 5C: Taxa whose relative abundance was impacted through supplementation of the Propionate-producing consortium (PPC) in the luminal compartment of the M-SHIME at the end of the stabilisation phase (time point 1), after clindamycin supplementation (time point 2), after 3 days of PPC administration (time point 3) and after 4 days of washout (time point 4). NS= not significantly different. Different superscripts indicate significantly different means across time points within a treatment.

Taxon	Time point	Treatment (Mean ± SEM)		Effect		
		PPC	Control	Time	Treatment	Time*Treatment
Anaerostipes	1	5.8E-7 ± 3.3E-7^{a}	1.2E-6 ± 7.8E-7^{a}	<0.0001	NS	NS
	2	2.4E-9 ± 1.1E-9^{b}	1.4E-9 ± 5.8E-10^{b}			
	3	2.2E-9 ± 9.2E-10^{b}	1.4E-9 ± 5.7E-10^{b}			
	4	1.7E-6 ± 1.3E-6^{b}	1.9E-6 ± 1.0E-6^{c}			
Bifidobacterium	1	0.0002 ± 0.00004^{a}	0.0002 ± 0.0001^{a}	<0.0001	NS	NS
	2	0.00006 ± 0.00003^{b}	0.00004 ± 0.00003^{b}			
	3	0.00005 ± 0.00004^{b}	0.00001 ± 0.00001^{c}			
	4	0.0002 ± 0.0001^{a}	0.00008 ± 0.00006^{d}			
Bilophila	1	0.0003 ± 0.0004^{a}	0.0005 ± 0.00004^{a}	<0.0001	NS	NS
	2	0.00004 ± 0.00001^{b}	0.00003 ± 0.00001^{b}			
	3	0.0001 ± 0.00005^{b}	0.0002 ± 0.0005^{a}			
	4	0.0003 ± 0.00004^{a}	0.0003 ± 0.00005^{a}			
Erysipelatoclostridium	1	9.0E-9 ± 3.7E-9^{a}	2.3E-9 ± 9.3E-10^{a}	<0.0001	NS	NS
	2	1.4E-6 ± 1.3E-6^{b}	1.7E-6 ± 1.5E-6^{b}			
	3	5.2E-6 ± 3.4E-6^{b}	2.5E-6 ± 2.3E-6^{b}			
	4	1.2E-6 ± 6.7E-7^{c}	1.4E-6 ± 9.7E-7^{b}			
Escherichia-Shigella	1	0.0015 ± 0.0004^{a}	0.0027 ± 0.0014^{a}	NS	0.009	NS
	2	0.0026 ± 0.0002^{b}	0.0052 ± 0.001^{b}			
	3	0.0021 ± 0.0005^{b}	0.0064 ± 0.001^{b}			
	4	0.003 ± 0.001^{c}	0.0066 ± 0.001^{b}			
Flavonifractor	1	0.00002 ± 6.E-6^{a}	0.00002 ± 8.7E-6^{a}	0.009	NS	NS
	2	0.00001 ± 8.6E-6^{b}	0.00001 ± 7.7E-6^{b}			
	3	0.00004 ± 0.00001^{c}	0.00002 ± 0.00001^{b}			
	4	0.00003 ± 0.00001^{c}	6.3E-6 ± 3.5E-6^{a}			
Lachnoclostridium	1	0.0046 ± 0.0009^{a}	0.0055 ± 0.0011^{a}	0.02	NS	NS
	2	0.0055 ± 0.0011^{a}	0.0043 ± 0.0013^{b}			
	3	0.0056 ± 0.0015^{b}	0.0060 ± 0.0018^{ac}			
	4	0.0045 ± 0.0013^{ab}	0.0040 ± 0.0011^{b}			
Parabacteroides	1	0.0012 ± 0.0003^{a}	0.0011 ± 0.0002^{a}	<0.0001	NS	NS
	2	0.0047 ± 0.0014^{b}	0.0041 ± 0.0005^{b}			
	3	0.0031 ± 0.0006^{bc}	0.0025 ± 0.004^{c}			

	4	0.0031 ± 0.0005^{c}	0.0023 ± 0.0004^{c}			
Unclassified Enterobacteriaceae	1	0.0006 ± 0.0001^{a}	0.0003 ± 0.0001^{a}	<0.0001	NS	NS
	2	0.0019 ± 0.0003^{b}	0.0019 ± 0.0003^{b}			
	3	0.0018 ± 0.0005^{b}	0.0022 ± 0.0005^{b}			
	4	0.0012 ± 0.0006^{c}	0.0020 ± 0.0003^{b}			
Unclassified Lachnospiraceae	1	1.3E-9 ± 0.0003^{a}	1.0E-13 ± 2.6E-8^{a}	NS	0.02	NS
	2	0.0003 ± 0.00009^{b}	0.0002 ± 0.00003^{b}			
	3	0.0005 ± 0.00009^{b}	0.0003 ± 0.0001^{b}			
	4	0.0011 ± 0.0003^{c}	0.0001 ± 0.0002^{b}			
Unclassified Ruminococcaceae	1	0.00001 ± 0.00001^{a}	0.00004 ± 0.00002^{a}	<0.0001	NS	NS
	2	5.5E-6 ± 3.2E-6^{b}	6.3E-7 ± 2.2E-7^{b}			
	3	3.3E-6 ± 1.1E-6^{b}	2.7E-6 ± 1.9E-7^{b}			
	4	7.5E-6 ± 3.4E-6^{c}	4.9E-6 ± 2.9E-6^{b}			

Supplementary Table 5D: Taxa whose relative abundance was impacted through the supplementation of the Propionate-producing consortium (PPC) in the mucosal compartment of the M-SHIME, at the end of the stabilisation phase (time point 1), after clindamycin supplementation (time point 2), after 3 days of PPC administration (time point 3). NS= not significantly different. Different superscripts indicate significantly different means across time points within a treatment.

Taxon	Time point	Treatment (Mean ± SEM)		Effect		
		PPC	Control	Time	Treatment	Time*Treatment
Alistipes	1	0.0078 ± 0.0003[a]	0.00278 ± 0.0007[a]			
	2	0.0044 ± 0.0003[a]	0.00146 ± 0.0007[a]	0.003	0.03	NS
	3	0.0002± 0.00006[b]	0.00006 ± 0.0002[b]			
Bifidobacterium	1	0.00053 ± 0.0014[a]	0.00028 ± 0.0014			
	2	0.00014 ± 0.0005[b]	0.00092 ± 0.00005	<0.0001	NS	NS
	3	0.00017 ± 0.0001[b]	0.00007 ± 0.00002			
Bilophila	1	0.0013 ± 0.0002[a]	0.0017 ± 0.0004[a]			
	2	0.00006 ± 0.0002[ab]	0.00003 ± 0.00004 [b]	<0.0001	NS	NS
	3	0.0006 ± 0.0001[b]	0.0006 ± 0.0002[c]			
Erysipelatoclostridium	1	0.0007 ± 0.0001[a]	0.0007 ± 0.0001[a]			
	2	0.0027 ± 0.001[ab]	0.0050 ± 0.0009 [b]	<0.0001	0.003	NS
	3	0.0011 ± 0.0002[b]	0.0009 ± 0.0002[c]			
Parabacteroides	1	0.0007 ± 0.0001[a]	0.0007 ± 0.0001[a]			
	2	0.0027 ± 0.001[ab]	0.0050 ± 0.0009 [b]	<0.0001	NS	NS
	3	0.0011 ± 0.0002[b]	0.0009 ± 0.0002[c]			
Unclassified Enterobacteriaceae	1	0.0021 ± 0.0007[a]	0.0011 ± 0.0003[a]			
	2	0.0033 ± 0.0011[ab]	0.0025 ± 0.0006[b]	<0.0001	NS	NS
	3	0.0055 ± 0.0010[b]	0.0074 ± 0.0017[c]			
Unclassified Lachnospiraceae	1	0.0014 ± 0.0002[a]	0.0010 ± 0.0002[a]			
	2	0.00007 ± 0.0003[ab]	0.0004 ± 0.00005[b]	0.0006	0.01	NS
	3	0.0005 ± 0.0002[b]	0.0002 ± 0.00005[c]			
Unclassified Lactobacillaceae	1	1.74E-8 ± 7.12E-9[a]	4.3E-12 ± 2.68E-12[a]			
	2	1.68E-8 ± 6.6E-9[b]	4.1E-12 ± 2.5E-12[a]	<0.0001	<0.0001	<0.0001
	3	0.0002 ± 3.9E-6[c]	1.6E-8 ± 6.1E-9[b]			

Supplementary Table 6: Differences in absolute abundances of the certain genera included in the propionate-producing consortium between control and treatment vessels in the lumen compartment. The significant increase in Veillonella in the treatment vessels indicates its successful direct engraftment (P<0.05). The differences were assessed by 2-way Anova test using Holm-sidak method with significance at P<0.05. Time point (TPT) indicates the different phases at which the samples were analysed (1: End of stabilization phase, 2: After antibiotic treatment, 3: After 3 doses of propionate producing consortium, and 4: Washout phase).

**Genus**	**TPT**	CTR (Mean % ± SEM)	TRT (Mean % ± SEM)	***P* value**
Veillonella	1	0	0	NS
	2	0	0	NS
	3	0	4.95^{e}+06 ± 7.56e+05	<0.0001
	4	0	4.09e+04 ± 1.98e+04	NS
Akkermansia	1	1.76e+07 ±1.13e+07	6.75e+07 ± 4.85e+07	NS
	2	0	1.14e+04 ± 1.14e+04	NS
	3	5.69e+06 ± 4.28e+06	9.47e+04 ± 7.44e+07	NS
	4	1.21e+08 ±7.71e+07	1.45e+08 ± 7.67e+07	NS

Supplementary Table 7: Differences in absolute abundances of the certain genera included in the propionate-producing consortium between control and treatment vessels in the mucin compartment. The significant increase in Veillonella in the treatment vessels indicates its successful direct engraftment (P<0.05). The differences were assessed by 2-way Anova test using Holm-sidak method with significance at P<0.05. Time point (TPT) indicates the different phases at which the samples were analysed (1: End of stabilization phase, 2: After antibiotic treatment, and 3: After 3 doses of propionate producing consortium).

**Genus**	**TPT**	CTR (Mean % ± SEM)	TRT (Mean % ± SEM)	***P* value**
Veillonella	1	3.80e+04 ± 2.56e+04	8.57e+03 ± 8.57e+03	NS
	2	0	0	NS
	3	0	2.66e+06 ± 8.69e+05	<0.0001
Akkermansia	1	1.13e+07 ± 6.66e+06	9.95e+06 ± 4.64e+06	NS
	2	1.22e+06 ± 5.78e+05	7.28e+06 ± 5.48e+06	NS
	3	2.36e+07 ± 2.21e+07	7.60e+07 ± 5.88e+07	NS

Supplementary Table 8: Differences in absolute abundances of the certain genera that were involved in propionate production after adding the propionate-producing consortium in the lumen compartment. The significant increase in unclassified Lactobacillacea and unclassified Lachnospiraceae in the treatment vessels indicates successful indirect reinforcement of those genera (P<0.05). The differences were assessed by 2-way Anova test using Holm-sidak method with significance at P<0.05. Time point (TPT) indicates the different phases at which the samples were analysed (1: End of stabilization phase, 2: After antibiotic treatment, 3: After 3 doses of propionate producing consortium, and 4: Washout phase).

**Genus**	**TPT**	CTR (Mean % ± SEM)	TRT(Mean % ± SEM)	***P* value**
unclassified_Lactobacillacea	1	0	0	NS
	2	0	0	NS
	3	0	5.41e+05 ± 1.44e+05	<0.0001
	4	3.50e+04 ± 3.50e+04	6.11e+04 ± 2.77e+04	NS
unclassified Lachnospiraceae	1	4.16e+07 ± 8.25e+06	4.85e+07 ± 7.45e+06	NS
	2	5.98e+06 ± 1.42e+06	5.51e+06 ± 1.24e+06	NS
	3	1.66e+07 ± 6.82e+06	2.26e+07 ± 6.12e+06	NS
	4	9.56e+06 ± 1.62e+06	3.45e+07 ± 1.03e+07	0.0318
Bilophila	1	2.97e+07 ± 4.18e+06	2.99e+07 ± 3.43e+06	NS
	2	1.30e+06 ± 8.49e+05	2.62e+06 ± 1.40e+06	NS
	3	1.11e+07 ± 3.50e+06	1.05e+07 ± 4.03e+06	NS
	4	1.96e+07 ± 3.75e+06	2.27e+07 ± 3.02e+06	NS

Supplementary Table 9: Differences in absolute abundances of the certain genera that were involved in propionate production after adding the propionate-producing consortium in the mucin compartment. The significant increase in unclassified Lactobacillacea in the treatment vessels indicates successful indirect reinforcement of this genus in the mucin ($P<0.05$). The differences were assessed by 2-way Anova test using Holm-sidak method with significance at $P<0.05$. Time point (TPT) indicates the different phases at which the samples were analysed (1: End of stabilization phase, 2: After antibiotic treatment, and 3: After 3 doses of propionate producing consortium).

**Genus**	**TPT**	CTR (Mean % ± SEM)	TRT(Mean % ± SEM)	***P value***
unclassified_Lactobacillacea	1	0	0	NS
	2	0	0	NS
	3	0	7.27e+05 ± 1.91e+05	<0.0001
unclassified Lachnospiraceae	1	4.21e+07 ± 8.90e+06	5.93e+07 ± 1.69e+07	NS
	2	1.01e+07 ± 2.32e+06	2.37e+07 ± 8.12e+06	NS
	3	9.44e+06 ± 3.30e+06	1.85e+07 ± 8.62e+06	NS
Bilophila	1	5.37e+07 ± 1.39e+07	6.35e+07 ± 1.48e+07	NS
	2	8.73e+06 ± 2.38e+06	1.91e+07 ± 7.24e+06	NS
	3	1.70e+06 ± 6.18e+06	1.67e+07 ± 4.23e+06	NS

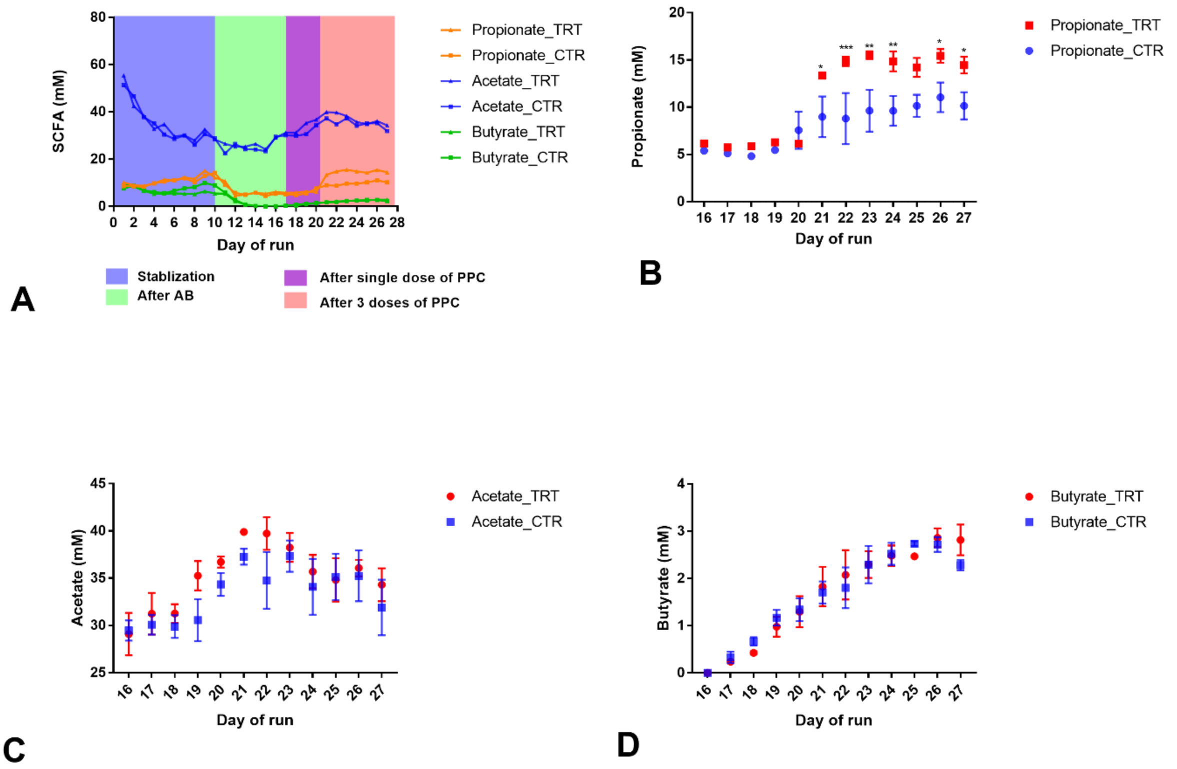

Supplementary Figure 1: Addition of the propionate producing consortium (PPC) promotes recovery of propionate production after antibiotic-associated disruption in donor 1. (A) Short chain fatty acid production during the 4 different phases of the experiment: Stabilization, post-antibiotic disruption, post-addition of a single dose of the propionate producing consortium, and post-addition of 3 doses of the propionate producing consortium. Single dose of treatment was added on day 17, and the 3 doses were added on days 20, 21, and 22. (B) Propionate was the main short chain fatty acid impacted already after 2 doses (Days 21-27) in the treatment reactors ($P < 0.05$). No significant difference was detected for acetate (C) and butyrate (D) after the treatment was added.

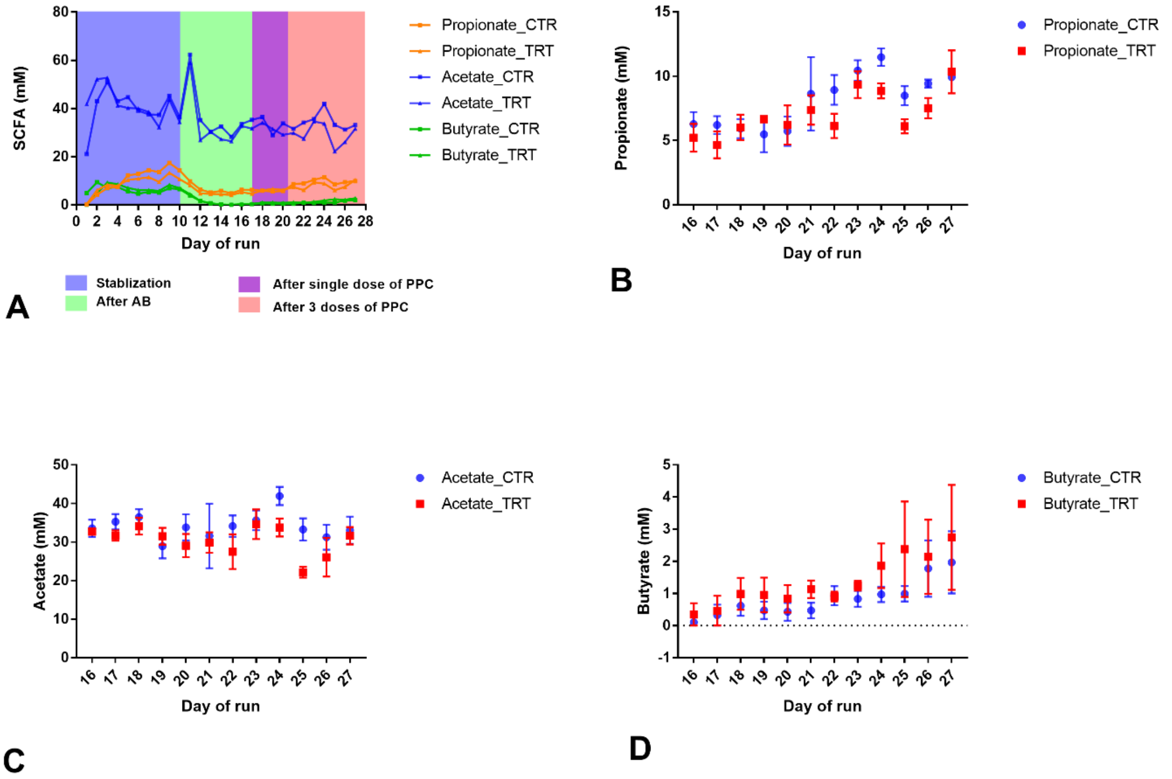

Supplementary Figure 2: Addition of the propionate producing consortium (PPC) did not have any significant effect propionate production after antibiotic-associated disruption in donor 2. (A) Short chain fatty acid production during the 4 different phases of the experiment: Stabilization, post-antibiotic disruption, post-addition of a single dose of the propionate producing consortium, and post-addition of 3 doses of the propionate producing consortium. Single dose of treatment was added on day 17, and the 3 doses were added on days 20, 21, and 22. (B) Propionate was the main short chain fatty acid was not impacted after any of the treatment doses (Days 21-27) in the treatment reactors (*P* >0.05). No significant difference was detected for acetate (C) and butyrate (D) after the treatment was added.

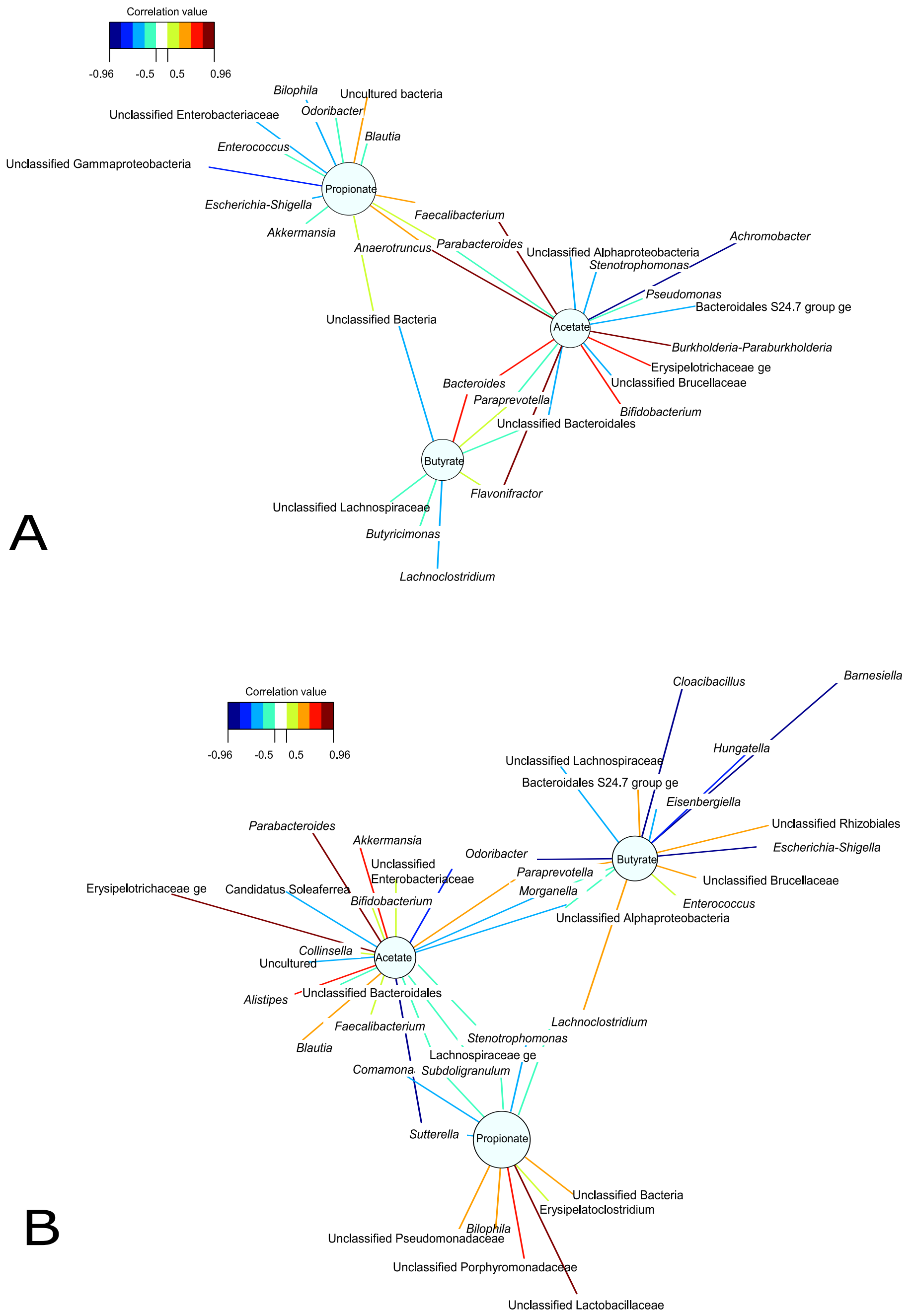

Supplementary Figure 3: Network of bacterial interactions influencing the production of the major short chain fatty acids over time. (A) Treatment reactors after antibiotics treatment and before adding 3 doses of the propionate consortium, (B) Treatment reactors after 3 days period of washout after the propionate consortium dosing. These bipartite networks are based on the regularised canonical correlations between relative bacterial abundances and relative concentrations of the main SCFA. Interactions have been filtered for an absolute correlation above 0.8 and are coloured following the key shown. Significant interactions are shorter lines, and genera with similar abundances within SHIME compartment tend to cluster closely. Networks depict genera potentially performing roles in propionate production.

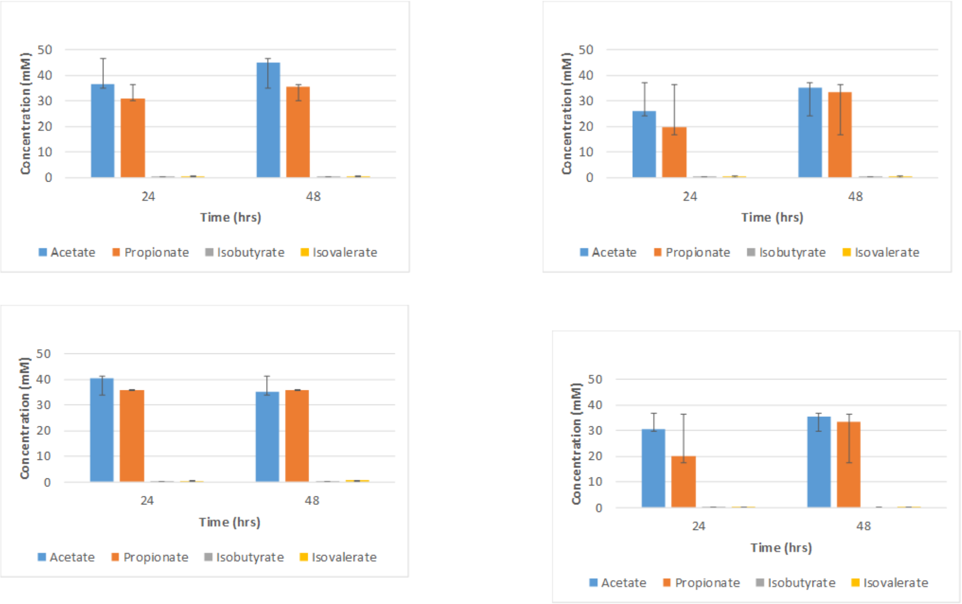

Supplementary Figure 4: Production of SCFA after 48h of a fed batch for the 7 strains of the propionate producing consortium. One dose of the consortium was prepared fresh on a daily basis for each day of the treatment on the SHIME run. Four replicates of the fed batch are presented with an average of 34.5 mM.

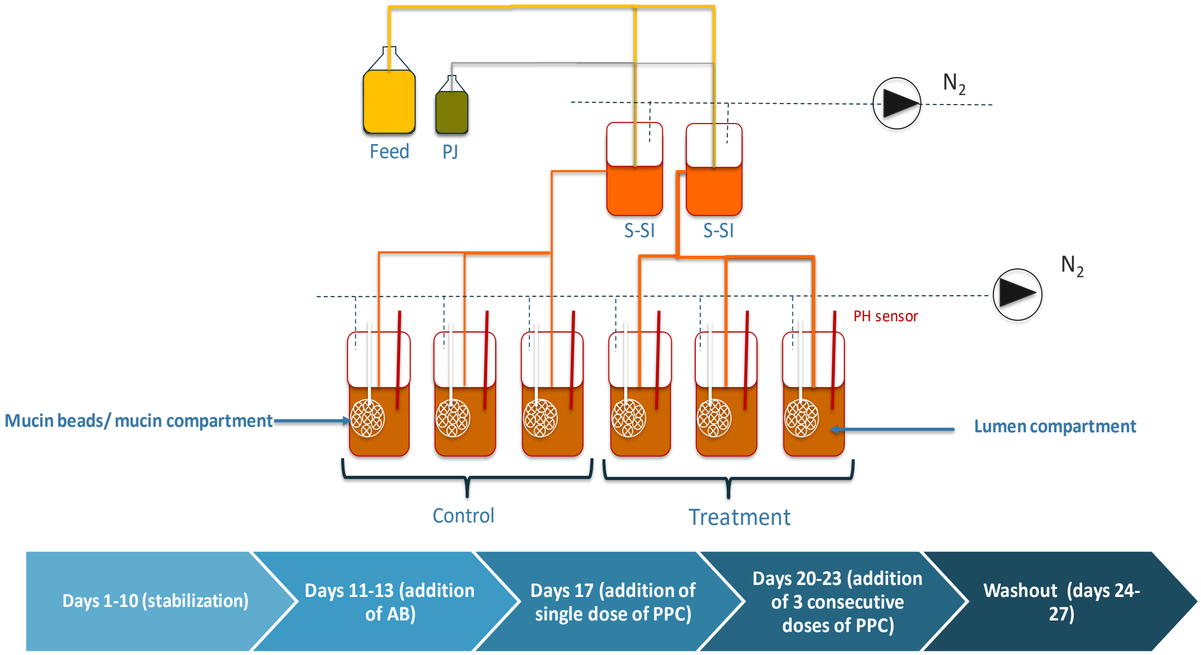

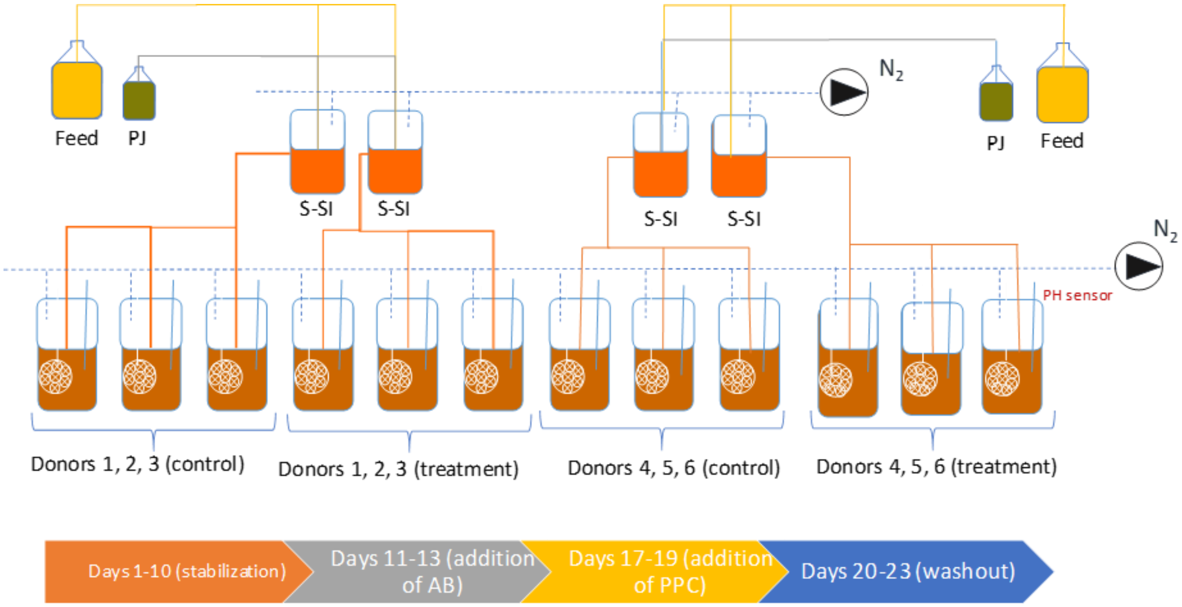

Supplementary Figure 5: Scheme of SHIME setups used in our experiments with the timeline of each experiment. M-SHIME setup for the single-donor experiment in which the fecal sample from one donor was tested at a time. Control and treatment vessels were in triplicates (A). A timeline is provided under the SHIME setup showing the different phases of the SHIME run at different days for the total of 27 days (A). M-SHIME setup for the multiple-donor experiment in which the fecal samples from six different donors were inoculated in separate vessels considering control and treatment condition as shown in (B). A timeline is provided under the SHIME scheme showing the different phases of the SHIME run at different days for the total of 23 days (B).

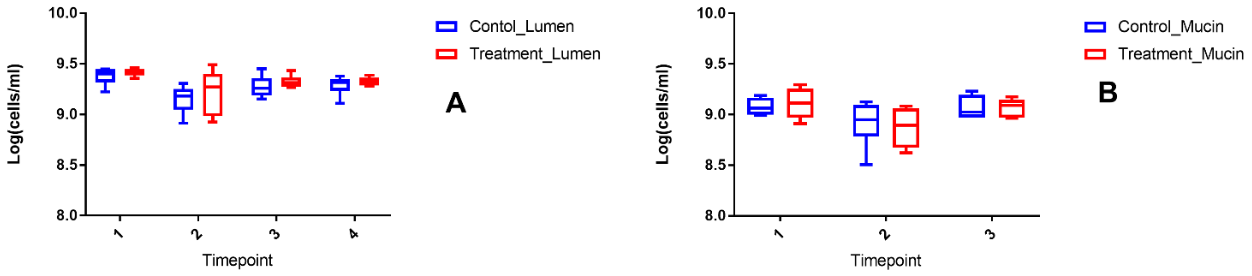

Supplementary Figure 6: Cell counts were significantly lower after antibiotic use in both lumen and mucin compartments (P<0.05). Cell counts for lumen SHIME samples significantly decreased after antibiotic treatment (P<0.05). Cell counts for lumen samples were compared at 4 different time points for 4 different SHIME phases: 1-Stabilization phase, 2-after antibiotic phase, 3-after PPC treatment phase, and 4-the last day of the SHIME run that is the washout phase. There was no significant increase for the cell load after PPC treatment (A). Cell counts for mucin SHIME samples significantly decreased after antibiotic treatment (P<0.05). Cell counts for mucin samples were compared at 4 different time points for 3 different SHIME phases: 1-Stabilization phase, 2-after antibiotic phase, and 3-after PPC treatment phase. There was no significant increase for the cell load after PPC treatment (B).

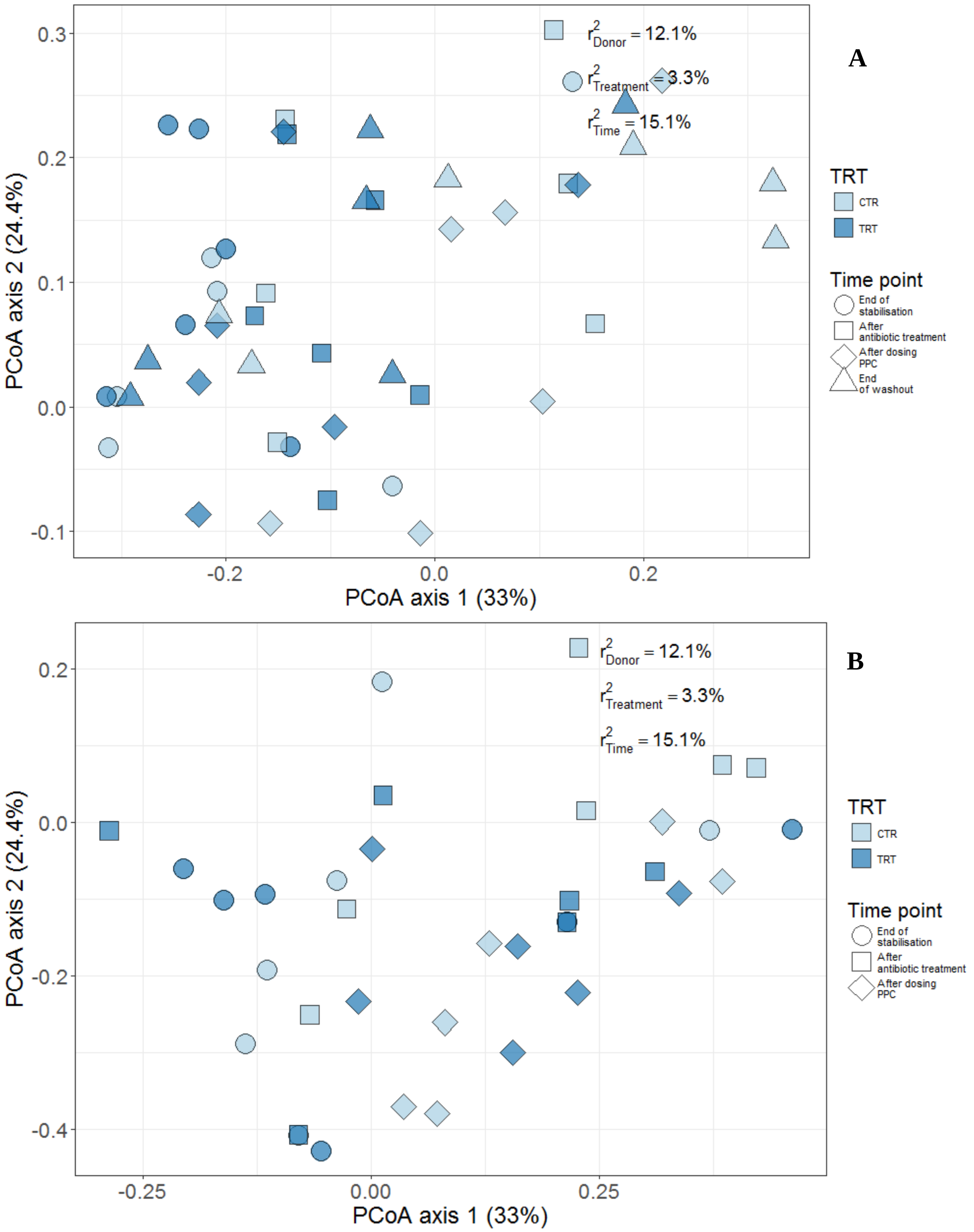

Supplementary Figure 7: PCoA analysis showing the dispersion of the microbial community by treatment for the 6 donors in the lumen (A) and mucin (B) compartments.
